# N-acetylcysteine Protects Against Myocardial Ischemia–Reperfusion Injury Through Anti-ferroptosis in Type 1 Diabetic Mice

**DOI:** 10.1007/s12012-024-09852-7

**Published:** 2024-04-22

**Authors:** Dongcheng Zhou, Yuhui Yang, Jiajia Chen, Jiaqi Zhou, Jianfeng He, Danyong Liu, Anyuan Zhang, Bixian Yuan, Yuxin Jiang, Weiyi Xia, Ronghui Han, Zhengyuan Xia

**Affiliations:** 1 Department of Anesthesiology, Affiliated Hospital of Guangdong Medical University, Zhanjiang, China; 2 State Key Laboratory of Pharmaceutical Biotechnology, Department of Medicine, The University of Hong Kong, Pok Fu Lam Road, Hong Kong

**Keywords:** Diabetes mellitus, Myocardial ischemia reperfusion injury, Ferroptosis, Lipid peroxidation, NAC

## Abstract

The hearts of subjects with diabetes are vulnerable to ischemia–reperfusion injury (IRI). In contrast, experimentally rodent hearts have been shown to be more resistant to IRI at the very early stages of diabetes induction than the heart of the non-diabetic control mice, and the mechanism is largely unclear. Ferroptosis has recently been shown to play an important role in myocardial IRI including that in diabetes, while the specific mechanisms are still unclear. Non-diabetic control (NC) and streptozotocin-induced diabetic (DM) mice were treated with the antioxidant N-acetylcysteine (NAC) in drinking water for 4 week starting at 1 week after diabetes induction. Mice were subjected to myocardial IRI induced by occluding the coronary artery for 30 min followed by 2 h of reperfusion, subsequently at 1, 2, and 5 week of diabetes induction. The post-ischemic myocardial infarct size in the DM mice was smaller than that in NC mice at 1 week of diabetes but greater than that in the NC mice at 2 and 5 week of diabetes, which were associated with a significant increase of ferroptosis at 2 and 5 week but a significant reduction of ferroptosis at 1 week of diabetes. NAC significantly attenuated post-ischemic ferroptosis as well as oxidative stress and reduced infarct size at 2 and 5 week of diabetes. Application of erastin, a ferroptosis inducer, reversed the cardioprotective effects of NAC. It is concluded that increased oxidative stress and ferroptosis are the major factors attributable to the increased vulnerability to myocardial IRI in diabetes and that attenuation of ferroptosis represents a major mechanism whereby NAC confers cardioprotection against myocardial IRI in diabetes.

**Supplementary Information:**

The online version contains supplementary material available at 10.1007/s12012-024-09852-7.

## Introduction

Diabetes and its complications are severe threats to human health around the world [1–4]. Ischemic heart disease is one of the most serious complications in patients with diabetes mellitus (DM), increasing morbidity and mortality [5]. The best way to save the ischemic myocardium is to restore blood flow to the ischemic heart. However, reperfusion itself may lead to further exacerbation of myocardial cell death, a phenomenon known as myocardial ischemia–reperfusion injury (MIRI) [6, 7]. Several potential mechanisms that form the basis of MIRI pathogenesis include mitochondrial dysfunction, oxidative stress, and calcium overload [8–10]. Clinical studies have shown that diabetes increases the susceptibility to MIRI [6, 11]. DM makes the heart vulnerable to IRI, but it can also cancel or impair the effectiveness of cardioprotective interventions, such as ischemic preconditioning, postconditioning, and pharmacologic preconditioning that are otherwise effective in subjects without diabetes [12–14]. The available clinical evidence strongly supports that DM increases cardiac susceptibility to IRI [14].

In a previous study, we reported increased myocardial oxidative stress, reduced antioxidant capacity, and impaired activation of the pro-survival proteins Akt and STAT3 in the myocardium of streptozotocin (STZ)-induced diabetic rats, which was accompanied with the diabetic heart increased sensitivity to IRI at 5 week after diabetes induction [15]. Despite a wealth of clinical data available that also suggest that diabetic hearts are more sensitive to IRI than the hearts of non-diabetic subjects, some in vivo studies in diabetic animal models have shown conflicting results, such as either increased or reduced sensitivity to myocardial ischemia with or without reperfusion injury [14, 16–19]. These discrepancies may be attributable to the duration and severity of diabetes in animal models [20]. However, the questions are, what is the exact time point during the progression of diabetes at which the diabetes heart becomes more sensitive to IRI relative to that of non-diabetic subjects, and what the underlying mechanism is? Answering these questions should have clinical importance, which will facilitate the development of new therapeutic strategies to mitigate myocardial injury. However, a study that addresses and compares the impact of the duration of diabetic status on myocardial sensitivity to IRI systemically in a single report is lacking, not to mention the related mechanistic exploration in this context.

Ferroptosis is a newly identified form of cell death caused by iron-dependent and reactive oxygen species (ROS)-induced lipid peroxidation [21]. Biochemically, intracellular glutathione (GSH) depletion, reduced glutathione peroxidase 4 (Gpx4) activity and solute carrier family 7, member 11(Slc7a11) deficiency occurred during the development of ferroptosis. Doxorubicin-induced myocardial injury or ischemia/reperfusion-induced myocardial injury was found to be strongly associated with ferroptosis, and selective inhibition of myocardial ferroptosis attenuated myocardial injury [22]. Gao et al. found that inhibition of glutamine catabolism (an important component of ferroptosis) reduced MIRI [23]. In the MIRI rat model and in oxygen–glucose deprivation/reoxygenation (OGD/R) H9C2 cells, acyl-CoA synthetase long-chain family member 4 (ACSL4)-mediated ferroptosis was found to be a promising target for MIRI therapy [24]. Ferroptosis has been shown to be associated with diabetic MIRI [25–28]. However, the exact impact of the extent of ferroptosis on diabetic heart vulnerability to MIRI is largely unknown.

Various studies have shown that N-acetylcysteine (NAC), an antioxidant, can not only reduce the overproduction of ROS in the heart of diabetic mice, but also reduce MIRI and improve cardiac function in diabetic mice after ischemia [29–34]. However, whether or not NAC mediated protection against MIRI in diabetes is attributable to the inhibition of ferroptosis is unclear. In this study, we found that cardiac levels of ferroptosis at 1 week of diabetes (D1w) did not significantly change or even tended to reduce, while cardiac ferroptosis started to increase at 2 week of diabetes (D2w) and significantly increased at 5 week of diabetes (D5w). Increased oxidative stress and ferroptosis could be the major factors attributable to the increased myocardial susceptibility to IRI in diabetes.

## Materials and Methods

### Animal Model Establishment and Drug Administration

Male C57BL/6J mice (weighing 20 ± 5 g, at the age of 8 week) obtained from Guangdong Medical Laboratory Animal Center were used in this study and acclimated in cages for 5 days prior to the experiment. The maximum number of mice per cage was limited 2–3 mice with the aim to optimize the environment of free movement and access to water and food. All mice were kept in captivity and received standard mouse food with free access to water according to the principles of animal care at Guangdong Medical University. The use of animals for this study was approved by the Committee on the Use of Live Animals in Teaching and Research of Guangdong Medical University.

The diabetes model was established by injecting Streptozotocin (STZ, freshly dissolved in 0.1 M citrate buffer, pH 4.5, Solarbio, China) intraperitoneally at the dose of 50 mg/kg per day for 5 consecutive days, while control mice (*n* = 8 per group) were injected with equal volume of citrate buffer. 3 days after STZ injection, glucose levels were measured using a glucose analyzer (Beckman Instruments, Fullerton, CA, USA) and mice with hyperglycemia (plasma glucose ≥ 16.7 mM) were considered diabetic and used for the ensuing experiments. The ferroptosis inducer Erastin (20 mg/kg) was administered intraperitoneally in 5 week diabetic mice before inducing myocardial ischemia reperfusion [28].

### Ultrasonographic Evaluation of Mouse Heart Function

A Visual Sonics Vevo 2100 high-resolution small animal ultrasound system (probe frequency: 40 MHz) was used for cardiac ultrasonography. The mice were placed in an airtight anesthesia induction chamber, filled with an oxygen gas mixture containing 3% isoflurane to rapidly induce anesthesia, and were removed when the mice were unable to turn freely, and fixed on the ultrasound table. At this point the concentration of isoflurane in the gas mixture is reduced to 1.5–2% to maintain mouse anesthesia. The mice were debrided on the chest, coated with an appropriate amount of ultrasound coupling agent, and two-dimensional ultrasound images were acquired at the long-axis and short-axis levels of the left ventricle, respectively, while satisfactory M-mode ultrasound images were obtained for preservation. The left ventricular ejection fraction (EF%) was measured on the M-mode ultrasound images using the analysis software provided with the ultrasound imaging system [35].

### Experimental Grouping

**Fig. 1:**
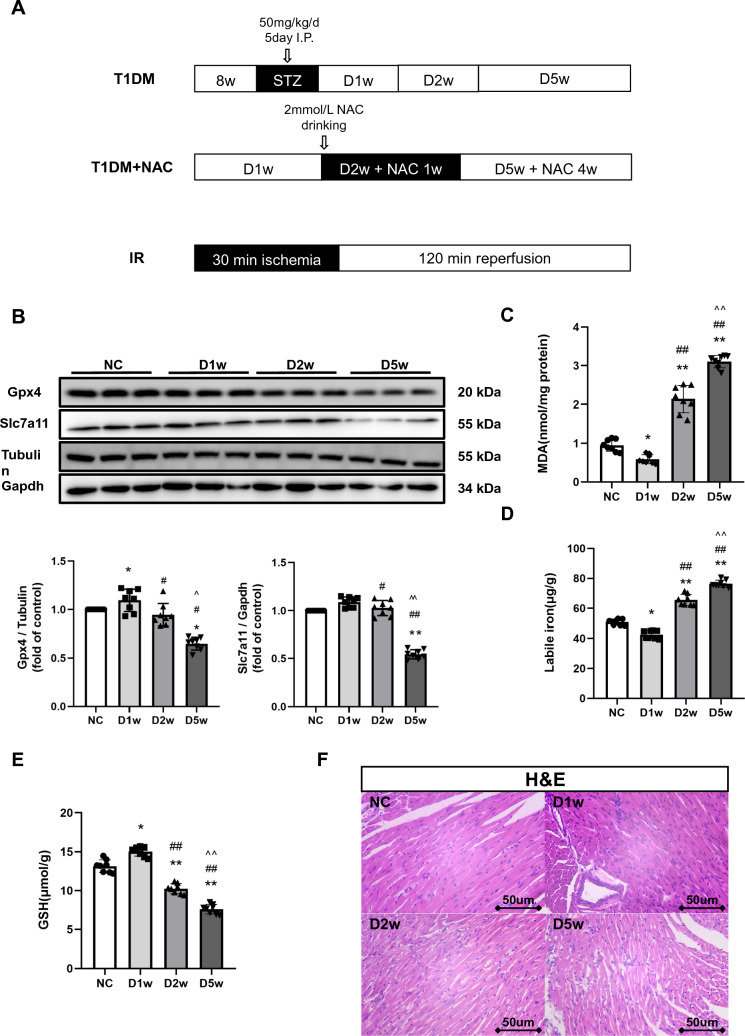
Diabetic heart’s time-dependent changes in Ferroptosis. **A** 8w mice were injected with STZ for at 50 mg/kg/d for 5 consecutive days to induce T1DM. DM mice were either untreated or started to receive NAC treatment at 1 week of T1DM for a duration of 1 week (D2w with NAC 1w) or for 4 week (D5w with NAC 4w) before being subjected to ischemia–reperfusion (IR) achieved by 30 min of coronary ligation followed by 2 h of reperfusion. **B** Expression of Gpx4 and Slc7a11 in mice with DM, assessed using Western blotting. Quantification of western blots was performed using Image J. **C** Heart tissue lipid peroxidation in DM mice, assessed by observing the changes in MDA levels. **D** Heart tissue labile iron levels in DM mice, assessed using the Iron Colorimetric Assay Kit. **E** Alterations of heart tissue GSH levels, assessed using the Glutathione Fluorometric Assay Kit. **F** H&E staining of myocardium, myofibrils with vacuolar degeneration, loose and lightly stained cytoplasm, inconspicuous transverse lines were observed in D5w. The magnifications is 40 times. Date are expressed as mean ± SD, *n* = 8 mice per group. *p < 0.05, **p < 0.01, versus NC group; ^#^*p* < 0.05, ^##^*p* < 0.01, versus D1w group; ^*p* < 0.05, ^^*p* < 0.01, versus D2w group. There is no significant statistical difference between groups without annotation symbols. *D1w* Diabetes for 1 week, *D2w* Diabetes for 2 week, *D5w* Diabetes for 5 week

All mice were equally randomly divided into four groups: Normal control (NC), Diabetes for a week (D1w), Diabetes for 2 week (D2w), Diabetes for 5 week (D5w). Thereafter, subgroups of diabetic mice received NAC treatment achieved by supplying NAC in drinking water at a concentration of 2 mmol/L for a duration of 1 week or 4 week, respectively, starting at 1 week of diabetes induction [36]. NAC (MedChemExpress, China) at this dose have no effect on GSH activity in non-diabetic control mice, but affected these parameters in diabetic mice [36]. Mice in NC group, D1w mice, D2w and D5w mice with or without NAC treatment were respectively subjected to MIRI after the completion of assigned treatments (Fig. 1A).

### Establishment of In Vivo MIRI and Assessment of Myocardial Infarct Size

Mice were anesthetized with an intraperitoneal injection of sodium pentobarbital at a dose of 80 mg/kg. After being anesthetized, the mice were placed in a supine position and secured on a dissection board, and an ECG monitor was installed. The left thoracic fur of the mice was shaved, and the shaved area was disinfected with alcohol-soaked cotton balls. A meticulous incision, about 1 cm in length, was delicately made on the area of skin where the pulsations of the heart were most distinctly palpable. The muscle layer was exposed, and the pectoralis major and pectoralis minor muscles were separated without damaging them. The pleura was then breached through the intercostal space between the third and fourth ribs, and then the heart was quickly squeezed out. Using 1–2 mm below the edge of the left atrial appendage and 0.5 mm beside the pulmonary artery cone as landmarks, a 6–0 silk suture was selected to ligate the left anterior descending (LAD) coronary artery. The needle was inserted to a depth of approximately 1 mm and a width of 1–2 mm to avoid piercing the heart. The ligature was tied in a slip knot, the proximal needle tip was trimmed short, and the distal end of the thread was left 3–4 cm outside the chest cavity. After ligation, the ventricular wall below the ligated area turned dark red or gray, and the heart was slowly pushed back into the chest cavity. Air was expelled from the chest cavity (to prevent pneumothorax), and the chest wall incision was closed with a purse-string suture, leaving the end of the ligation thread outside the chest for potential coronary recanalization. The ECG was closely monitored for changes within 30 min after ligation. Upon reaching the ischemic time, the ligature was slowly removed to restore the myocardial blood flow for 120 min.

The area of myocardial infarction was determined by Evans-Blue dye (Solarbio, China) and 2,3,5-triphenyltetrazolium chloride (TTC, Solarbio, China) double staining. The area in blue was considered as non-risk area. The area unstained by Evans Blue dye was identified as the area at risk (AAR), and the area unstained by TTC was considered as the infarcted tissue. Myocardial infarct size (IS) was calculated as the percentage of infarcted tissue divided by the AAR.

### Preparation of Myocardial Tissue Homogenate in Mice

After 30 min ischemia and 2 h reperfusion, the myocardial tissues were taken from mice. The tissue was rinsed repeatedly in pre-chilled saline to remove blood, filter paper was wiped dry, and weighed. Add saline at a ratio of 1:9 (W:V = 1 g:9 mL). The tissue homogenate was prepared in an ice-water bath. 3500 r/min centrifugation was performed and the supernatant was divided into EP tubes and stored in a refrigerator at – 80 ℃.

### Measurement of Plasma Creatine Kinase MB (CK-MB)

Plasma CK-MB levels were measured using a commercially available mouse ELISA kit (Ruixin Biology, China) as described [37].

### Determination of Malondialdehyde (MDA), Labile Iron, and Glutathione Peroxidase (GSH-PX) Activity in Tissues

Heart tissue MDA levels, labile iron levels, and GSH levels were measured using commercial MDA assay kit (Nanjing Jiancheng Bioengineering Institute, China), Iron Colorimetric Assay Kit (Cat#K390-100, BioVision, Milpitas, CA, USA), **GSH-PX** kit (Nanjing Jiancheng Bioengineering Institute, China), respectively, following the manufacturer's instructions [38].

### Determination of Plasma Levels of 15-F2t-Isoprostane

After 120 min of reperfusion, blood samples were collected from the root of the ascending aorta and then centrifuged to separate plasma for further measurements. 15-F2t-Isoprostane (15-F2t-IsoP) is a specific marker of lipid peroxidation, which was measured using an enzyme immunoassay kit (Cayman Chemical, Ann Arbor, MI) as we reported [29]. The absorbance was detected at 412 nm.

### Protein Extraction and Immunoblotting

Appropriate amount of left ventricular tissue was placed in a petri dish and chopped about 3 mm with cold Lysis Buffer [added 10 μl 100 × phosphatase inhibitor (AR1140 Boster, China), 10 μl 100 × protease inhibitor (AR1140 Boster, China), 10 μl 100 × EDTA (AR1140 Boster, China), 10 μl 100 mM PMSF (ST506, Beyotime, China) per 1 ml of cold Lysis Buffer (P0013, Beyotime, China)]. Then the tissue was grinded with a low temperature grinder to be homogenated until no obvious debris was visible and set aside on ice. The Lysis Buffer was sonicated and centrifuged at 12,000 rpm for 10 min at 4 ℃. Protein concentrations were determined using the Bradford assay (Beyotime, China). Equal amounts of the protein samples were separated on an SDS-PAGE gel and then transferred to a PVDF membrane. The membranes were blocked with 5% skim milk in Tris buffered saline (TBS)-Tween and incubated overnight at 4℃ with the following dilutions: Gpx4 (Catalog Number: 67763-1-Ig, Proteintech, China), Slc7a11 (Catalog Number: 26864-1 -AP, Proteintech, China) -AP, Proteintech, China), Ferritin (ab75973, Abcam, USA), Tubulin β (Catalog: AP0064, Bioworld Technology Inc, China). The membrane strips were washed with phosphate buffered saline (1xTBST) three times for 10 min each. After washing, the membrane strips were incubated with anti-rabbit IgG (A0208, Beyotime, China) and anti-mouse IgG (Catalog Number: SA00001-1, Proteintech, China), HRP-conjugated antibodies for 1 h at room temperature. After washing the membrane, the blots were developed with enhanced chemiluminescence reagent (UItraSignal, 4abio, China). Quantification of western blots was performed using Image J.

### Myocardial Tissue Morphological Assessment

An appropriate amount of left ventricular tissue was fixed with 4% paraformaldehyde for pathological examination. The morphological changes of myocardial tissue were observed by hematoxylin and eosin (HE) staining and Masson staining, respectively. Briefly, the 4% paraformaldehyde-fixed tissues were dehydrated in a gradient of ethanol concentration 70% → 80% → 90% → 95% → 100% chloroform transparent, wax dipped, paraffin embedded, and sectioned with a section thickness of about 3 μm. After dewaxing, the sections were stained with hematoxylin for 15 min and eosin for 3 min, respectively. Finally, they were observed under a microscope to assess the morphological changes of the heart. To assess whether fibrosis occurred at the heart lesion site, the sections were stained with Masson trichrome staining kit (Solarbio, China). Collagen fibers were stained blue, nuclei were stained black, and myocardium was stained red.

Prussian blue staining was performed to assess iron deposition in heart tissue according to the procedure provided in the commercial kit. After dewaxing, heart tissue sections were briefly incubated with Perls stain (equal volume mixture of reagent A and reagent B) (Solarbio, China) at 37 °C for 12 h. Then, the sections were washed 3 times with distilled water and immersed in Nuclear Solid Red solution for 15 min. Finally, the sections were rinsed, dehydrated, sealed, and observed under a microscope.

Transmission electron microscopy was performed using standard procedures of GDMS Biotechnology as we described [39]. Briefly, cells were fixed with an electron microscope fixative for 2–4 h. Cells were then embedded in 1% agarose, dehydrated, and cut into ultrathin sections (60–80 nm) using an ultrathin sectioning machine (Ultra-Microtome; Leica Microsystems GmbH, Wetzlar, Germany). Sections were stained with UO2 acetate in pure ethanol for 15 min, followed by lead citrate for 15 min. Images were obtained with a transmission electron microscope (JEM-1400; JEOL Ltd., Tokyo, Japan), and the images were taken using a Megaview III CCD camera (Soft Imaging System, Lakewood, CO, USA).

### Determination of the Expression of Ferritin by Immunohistochemistry

The cardiac tissue sections were deparaffinized in xylene and immersed in graded ethanol and distilled water. Immunohistochemical staining was performed using the avidin–biotin peroxidase complex (ABC) method according to the manufacturer’s instructions [40]. The sections were incubated with a rabbit monoclonal anti-ferritin antibody (1: 500, 4393S, CST, MA, USA). The positive areas of immunohistochemical staining were analyzed by Image-Pro Plus 6.0. The integrated optical density (IOD) was used in our research, which could exactly reflect the total protein expression in immunohistochemical staining.

### Assessment of Ferritin Gene Expression by Quantitative Real-Time PCR

At the end of the experimental period, mice are euthanized, and heart was immediately collected and preserved in RNA later solution to preserve the integrity of RNA. Total RNA was extracted using the TRIzol® reagent method and then reverse transcribed using a cDNA synthesis kit. Quantitative PCR was performed using the Roche Lightcycler 480 system with Roche SYBR Green Master Mix reagents (Roche Applied Science, Indianapolis, IN). The relative expression levels of genes of interest were determined using the 2-DeltaDeltaCt method, Actin served as an internal control. The sequences of all the specific primers were designed to span extron-intron to prevent the improper amplification of mRNA. The primer sequences were as follows: Ferritin, 5'-TGCCATCAACCGCCAGATCAAC-3' (Forward) and 5'-AGTTCTTCAGAGCCACATCATCTCG-3' (Reverse); Actin, 5'-CAGCAAGCAGCAGTACGATG-3' (Forward) and 5-'GCAGCTCAGTAACAGTCCG-3' (Reverse).

### Statistical Analysis

The statistical analyses were performed using GraphPad Prism 10.1.2 statistic software (La Jolla, CA). Data were presented as mean ± standard deviation (S.D.). For continuous variables, normal distribution was evaluated by the Shapiro–Wilk test. For comparisons in between two groups, Student-T test was performed with an adjusted *p* value of < 0.05 being considered statistically significant. For multiple groups, one-way ANOVA or a 2-way ANOVA was performed with post hoc Tukey multiple comparison tests with an adjusted *p* value of < 0.05 being considered statistically significant.

## Results

### Diabetic Heart Status of Ferroptosis at Various States of the Disease

To determine whether and when ferroptosis occurs in the diabetic heart, we established type 1 diabetes mellitus(T1DM) in mice and investigated cardiac ferroptosis at 1, 2, and 5 week of diabetes, as cardiac function was reported to be reduced at week-5 of T1DM [33]. As shown in Table 1, in STZ-induced diabetic mice, plasma glucose, water intake, and food consumption were significantly increased compared to non-diabetic mice. Gpx4 and Slc7a11 are the core indicators of ferroptosis pathway, their downregulation would lead to the occurrence of ferroptosis that is accompanied with labile iron upregulation. We found that the levels of cardiac Gpx4 started to increase as early as 1 week but dropped back to the original level at 2 week of diabetes (*p* < 0.05) (Fig. 1B). However, compared to the earlier changes in Gpx4, significant downregulation of Slc7a11 did not occur until at week-5 of diabetes (*p* < 0.05). Likewise, the levels of MDA (a lipid peroxidation marker) and labile iron levels increased in D2w (*p* < 0.05) that were concomitant with a decrease in GSH starting at 2 week of diabetes and onward (Fig. 1C–E). Interestingly, MDA and labile iron levels even decreased in the very first week of diabetes that was coincident with a transient increase of GSH, which means that the resistance to lipid peroxidation increased (all *p* < 0.05 vs. non-diabetic control). This may be related to the self-protection regulatory mechanism stimulated by the body in the beginning of the hyperglycemic environment. H&E staining of cardiac sections showed fewer myocardial cells, enlarged nucleus and increased intercellular space in the 5 week diabetic group and to a less degree in the 2 week diabetic group as compared with control group (Fig. 1F). Also, myofibrils with vacuolar degeneration, loose and lightly stained cytoplasm, inconspicuous transverse lines were observed in D5w. Generally, diabetes led to early changes in ferroptosis-related protein levels in the diabetic heart, with definite changes occurring by week-5, and the compensatory self-protection from diabetes 1 week possibly disappeared at 2 week of diabetes.

**Table 1:** General condition of normal, DM and NAC treated mice

Group	Body weight (g)	Food intake (g/d)	Water intake (ml/kg/d)	Blood glucose (mmol/L)
Normal control	25.14 ± 0.57	4.45 ± 1.01	315.43 ± 8.11	6.88 ± 1.03
D1w	23.64 ± 0.99*	8.53 ± 1.21*	709.40 ± 3.23 *	24.35 ± 1.52 *
D2w	20.49 ± 1.09*	8.62 ± 0.92 *	780.22 ± 5.51*	25.65 ± 0.78 *
D5w	20.64 ± 1.36*	9.13 ± 1.41 *	768.43 ± 4.91 *	27.71 ± 1.39 *
D2w + NAC	21.31 ± 1.00*#	7.71 ± 1.10 *#	753.12 ± 9.73 *	25.16 ± 1.27 *
D5w + NAC	22.40 ± 0.67*^	6.71 ± 0.52*^	711.41 ± 2.31 *^	21.22 ± 1.35 *^

All values are expressed as mean ± SD. *n* = 8 per group, water intake and food consumption values were the average value of corresponding weeks. Body weight and plasma glucose were measured on the day of execution or surgery

*D1w* Diabetes for a week, *D2w* Diabetes for 2 week, *D5w* Diabetes for 5 week, *D2w* + *NAC* Diabetes for 2 week and NAC treatment for 1 week, *D5w* + *NAC* Diabetes for 5 week and NAC treatment for 4 week

^*^p < 0.05 versus control, ^#^*p* < 0.05 versus D2w, ^*p* < 0.05 versus D5w

### Impact of the Duration of Diabetes on Myocardial Susceptibility to Ischemia Reperfusion and the Severity of Ferroptosis and Its Related Proteins

#### Diabetes at Week 1

**Fig. 2:**
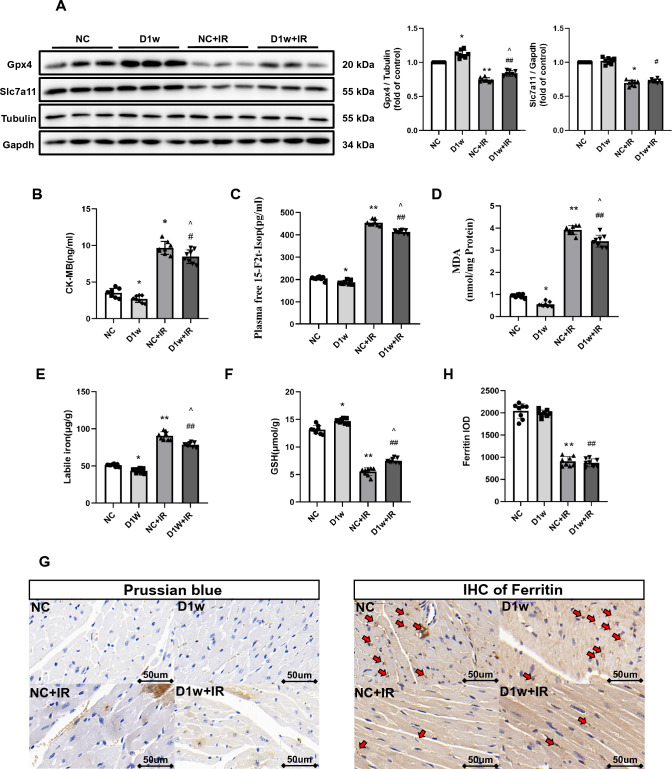
The MIRI in the first week of diabetic mice. **C** and **A** Expression of Gpx4 and Slc7a11 in mice with DM, assessed using Western blotting. **B** Levels of serum CK-MB, measured after reperfusion using the CK-MB ELISA kit. **C** and **D** Lipid peroxidation in DM mice, assessed by observing the changes in MDA and 15-F2t-IsoP levels. **E** Labile iron levels in DM mice, assessed using the Iron Colorimetric Assay Kit. **F** Alterations of GSH levels, assessed using the Glutathione Fluorometric Assay Kit. **G** Prussian blue stain showed abnormal iron deposition (brown) in myocardium, with the iron deposition increasing after IR, but little difference between D1w and control group. **H** Expression and the integrated optical density (IOD) of Ferritin assessed using IHC in myocardium (brown, arrow head) and, showing no difference between D1w + IR and NC + IR group. The magnifications is 40 times. Date are expressed as mean ± SD, *n* = 8 mice per group. *p < 0.05, **p < 0.01, versus NC group; ^#^*p* < 0.05, ^##^*p* < 0.01, versus D1w group; ^*p* < 0.05, ^^*p* < 0.01, versus NC + IR group. There is no significant statistical difference between groups without annotation symbols. *D1w* Diabetes for 1 week

We then used DM mice to establish a myocardial ischemia reperfusion (IR) model. After the impact of IR, ferroptosis related injury did happen in either the control or 1-week diabetic group. Notably, the degree of myocardial damage and ferroptosis in D1w mice were slightly but significantly attenuated, which was reflected as smaller infarct size, better cardiac function (Fig. 5), lower plasma CK-MB levels, 15-F2t-IsoP levels (a symbol of lipid peroxidation) and increased protein levels of Gpx4 and GSH (Fig. 2A–D, F). No significant cardiac morphological alteration was observed except for a slight increase in lipid droplets in D1w mice (Fig. 6). Ferritin degradation is known to increase intracellular free iron content and can activate ferroptosis. IHC results and labile iron levels also showed that abnormal iron infiltration has indeed been alleviated in D1w (Fig. 2E, H). These findings suggested that at the first week of diabetes, the heart is instead protected against ischemia–reperfusion injury, which is associated with a reduction in iron release in response to a transient increase in endogenous antioxidant capacity [41].

#### Diabetes at Week 2

**Fig. 3:**
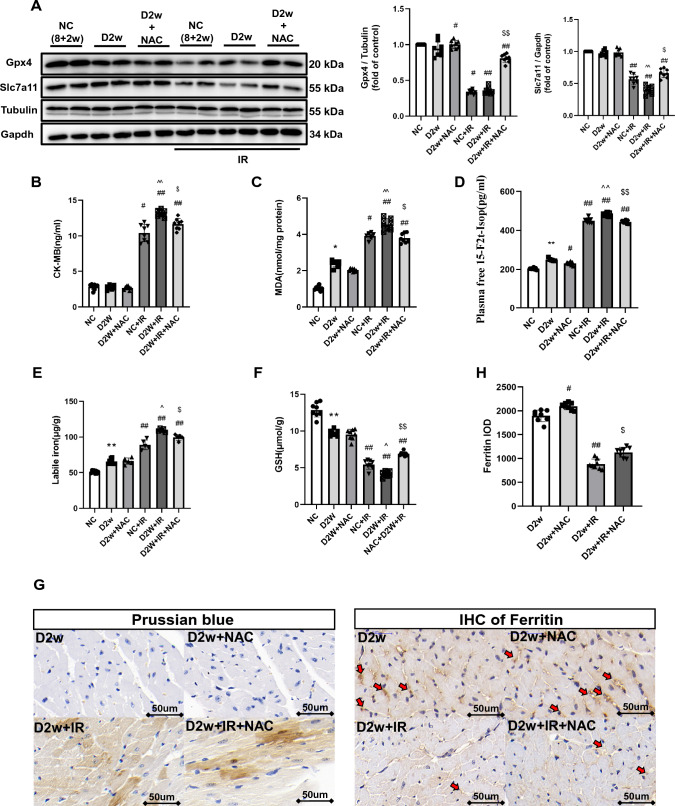
The MIRI with or without NAC treatment in second week of diabetic mice. **A** Expression of Gpx4 and Slc7a11 in mice with DM, assessed using Western blotting. **B** Levels of serum CK-MB, measured after reperfusion using the CK-MB ELISA kit. **C** and **D** Lipid peroxidation in DM mice, assessed by observing the changes in MDA and 15-F2t-IsoP levels. **E** Labile iron levels in DM mice, assessed using the Iron Colorimetric Assay Kit. **F** Alterations of GSH levels, assessed using the Glutathione Fluorometric Assay Kit. **G** Prussian blue stain showed abnormal iron (brown) deposition in myocardium. Iron infiltration was significantly increased after IRI in D2w mice, but there was no significant improvement after NAC treatment for 1 week. **H** Expression and the integrated optical density (IOD) of Ferritin assessed using IHC in myocardium (brown, arrowhead). Ferritin in D2w mice decreased significantly after IR, but it could still be detectable with IHC. Meanwhile, after NAC treatment, the ferritin levels were increased after ischemia by in D2w. The magnifications is 40 times. Data are expressed as mean ± SD, *n* = 8 mice per group. *p < 0.05, **p < 0.01, versus NC group; ^#^*p* < 0.05, ^##^*p* < 0.01, versus D2w group; ^*p* < 0.05, ^^*p* < 0.01, versus NC + IR group; ^$^*p* < 0.05, ^$$^*p* < 0.01, versus D2w + IR group. There is no significant statistical difference between groups without annotation symbols (*p* > 0.05). *D2w* Diabetes for 2 week, *D2w* + *NAC* Diabetes for 2 week and NAC treatment for 1 week

After 2 week of continuous hyperglycemia, at the baseline, there appeared to be more iron accumulation in the hearts of D2w mice than D1w (Fig. 3G). After suffering from IRI, the cardiac levels of Gpx4 and Slc7a11 were further downregulated, and post-ischemic infarct size was significantly bigger than that in non-diabetic control group (*p* < 0.05) (Fig. 3A, Fig. 5A). In D2w mice, cardiac GSH level was significantly reduced compared to non-diabetic controls (*p* < 0.05, D2w vs. NC, Fig. 3F) and was further decreased after IR (*p* < 0.05, D2w + IR vs. D2w, Fig. 3F) that was corresponded to significantly increased labile iron in the absence or presence of IR in D2w mice (Fig. 3E), which in combination with the increases in cardiac iron (Fig. 3G) and decreases in ferritin formation after IR (Fig. 3H) indicated that IR increased ferroptosis (*p* < 0.05). Lipid peroxidation levels was increased at D2w and were further increased after IR as confirmed by increased 15-F2t-IsoP and MDA levels (*p* < 0.05) (Fig. 3D, C). The increase of CK-MB also indicated that the acute myocardial injury was more severe in D2w mice than NC (*p* < 0.05) (Fig. 3B). No significant cardiac functional impairment was seen at D2w as compared to non-diabetic control mice (data not shown). As shown in the image photoed by transmission electron microscopy, the mitochondria of D2w mice were swollen with obscure and vacuolated ridges, and more lipid droplets were seen in D2w in the whole view (Fig. 6) than in D1w after IR. These results collectively suggest that after 2 week of developing diabetes mellitus, the heart began to show structural changes, and ferroptosis became obvious. And treatment with NAC for a duration of 1 week starting at 1 week after diabetes induction significantly enhanced post-ischemic levels of Slc7a11, Gpx4 (Fig. 3C) and GSH (Fig. 3F), decreased lipid peroxidation evidenced as reduction in MDA (Fig. 3C) and 15-F2t-Isoprostane (Fig. 3D) and reduced levels of labile iron (Fig. 3E), and attenuated post-ischemic ferroptosis and myocardial cellular damage (Fig. 3B).

#### Diabetes at Week 5

**Fig. 4:**
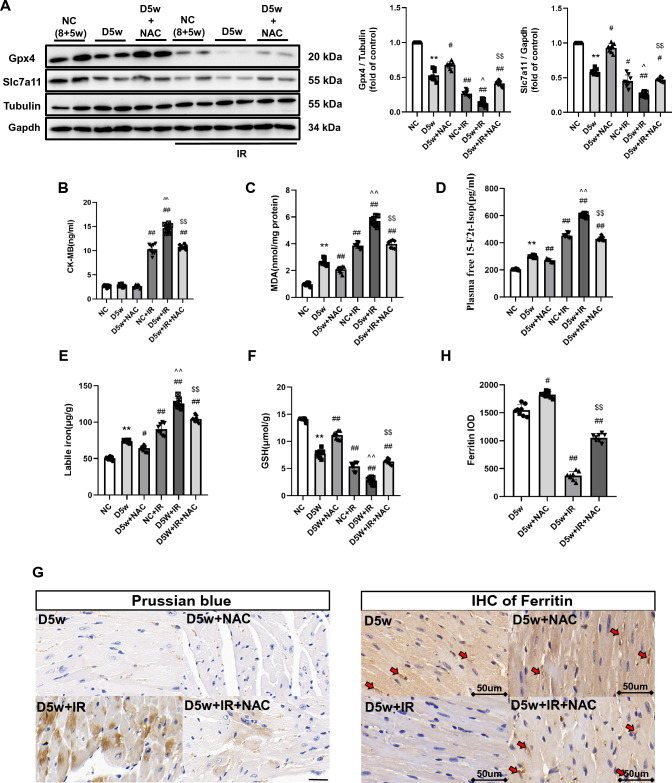
The MIRI with or without NAC treatment in the fifth week of diabetic mice. **A** Expression of Gpx4 and Slc7a11 in mice with DM, assessed using Western blotting. **B** Levels of serum CK-MB, measured after reperfusion using the CK-MB ELISA kit. **C** and **D** Lipid peroxidation in DM mice, assessed by observing the changes in MDA and 15-F2t-IsoP levels. **E** Labile iron levels in DM mice, assessed using the Iron Colorimetric Assay Kit. **F** Alterations of GSH levels, assessed using the Glutathione Fluorometric Assay Kit. **G** Prussian blue stain showed abnormal iron (brown) deposition in myocardium. Further increase in iron deposition was observed in D5w + IR group, while this enhancement of iron deposition was significantly reduced after 4 week of NAC treatment. **H** Expression of Ferritin assessed using IHC in myocardium (brown, arrowhead), Positive of Ferritin could hardly be detected after IR in D5w mice, but the degradation of Ferritin was significantly inhibited by NAC treatment for 4 week. The magnifications is 40 times. Date are shown as mean ± SD, *n* = 8 mice per group. *p < 0.05, **p < 0.01, versus NC group; ^#^*p* < 0.05, ^##^*p* < 0.01, versus D5w group; ^*p* < 0.05, ^^*p* < 0.01, versus NC + IR group; ^$^*p* < 0.05, ^$$^*p* < 0.01, versus D5w + IR group. There is no significant statistical difference between groups without annotation symbols (p > 0.05). *D5w* Diabetes for 5 week; *D5w* + *NAC* Diabetes for 5 week and NAC treatment for 4 week

**Fig. 5:**
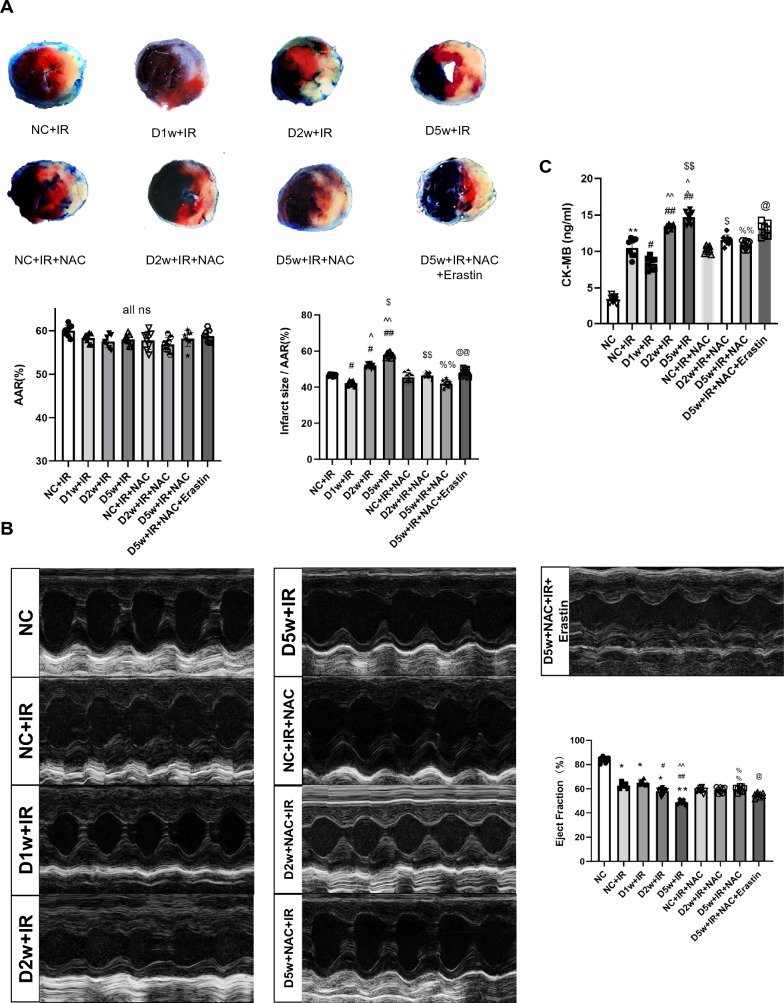
The effects of NAC on postischemic myocardial infract size and cardiac ultrastructure in diabetic mice by regulating Ferroptosis. **A** Infarct size (IS) is expressed as a percentage of the area at risk (AAR). Ischemia reperfusion (I/R) was achieved by 30-min ischemia followed by 2-h reperfusion in diabetic mices with or without NAC. **B** Cardiac ultrasound and changes of EF after IR. C Level of CK-MB showing the extent of myocardial damage. Date are expressed as mean ± SD, *n* = 8 mice per group. *p < 0.05, **p < 0.01, versus NC group; ^#^*p* < 0.05, ^##^*p* < 0.01, versus NC + IR group; ^*p* < 0.05, ^^*p* < 0.01, versus D1w + IR group; ^$^*p* < 0.05, ^$$^*p* < 0.01, versus D2w + IR group; ^%^*p* < 0.05, ^%%^*p* < 0.01, versus D5w + IR group; ^@^*p* < 0.05, ^@@^*p* < 0.01, versus D5w + IR + NAC group. There is no significant statistical difference between groups without annotation symbols (p > 0.05). *D1w* Diabetes for a week, *D2w* Diabetes for 2 week, *D5w* Diabetes for 5 week; *D2w* + *NAC* Diabetes for 2 week and NAC treatment for 1 week, *D5w* + *NAC* Diabetes for 5 week and NAC treatment for 4 week

**Fig. 6:**
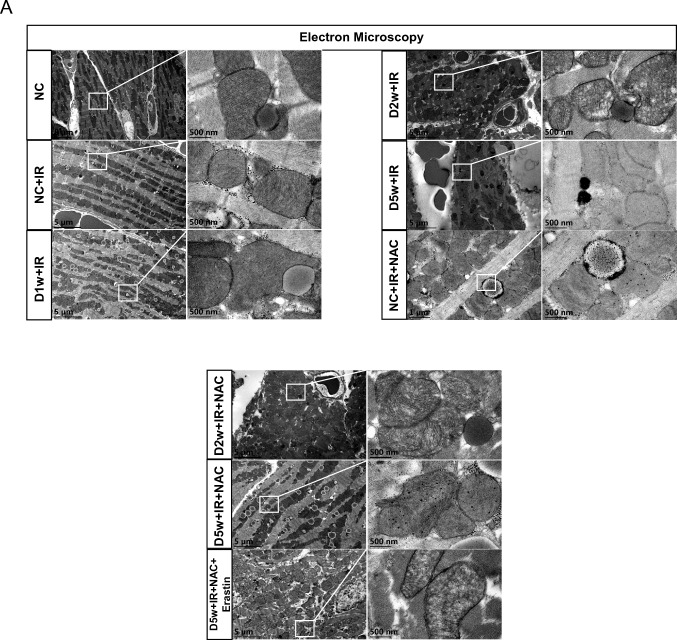
The effect of NAC on cardiac ultrastructure in diabetic mice with IR. **A** Typical morphological changes of ferroptosis in cardiomyocytes were observed using transmission electron microscopy. With the development of diabetes, ferroptosis-related injury during IR was gradually aggravated, which manifested as the increase and oxidation of lipid droplets in cardiac tissue, the disappearance of mitochondrial crest and the destruction of mitochondrial membrane increased. After 4 week of NAC treatment, mitochondrial damage was significantly alleviated. Erastin reverses the protective effect of NAC on cardiac ultrastructure. *D1w* Diabetes for a week, *D2w* Diabetes for 2 week, *D5w* Diabetes for 5 week, *D2w* + *NAC* Diabetes for 2 week and NAC treatment for 1 week, *D5w* + *NAC* Diabetes for 5 week with NAC treatment for 4 week

Next, we explored extent of myocardial IRI in D5w mice and its potential association with ferroptosis. As shown in Fig. 5A, the heart of D5w mice with IR reached the largest infarct size relative to that seen in the D2w and D1w groups (Fig. 5A). Prussian blue staining also showed more pronounced iron infiltration in the infarcted zones (Fig. 4G) compared to the heart of D2w with IR in Fig. 3G. The ratios of Gpx4 and Slc7a11 were significantly reduced to a very low level (Fig. 4A) and the IHC of ferritin also barely found positively stained (*p* < 0.05) (Fig. 4H). To further confirm the expression of ferritin, we performed western blotting and qPCR and found that both the mRNA and protein levels of ferritin were significantly decreased, which was in accordance with the IHC result (Supplementary Fig. 3A, B). Labile iron, 15-F2t-IsoP, and MDA levels further increased and GSH showed further depletion (*p* < 0.05) (Fig. 4C–F). The ultrastructure of cardiac tissue showed that the lipid droplets increased significantly and appeared black, which is indicative of a significant increase in lipid peroxidation. Low ejection fraction suggested that cardiac function was also severely impaired in D5w IR mice (Fig. 5B). Under a high-power microscope, most of the mitochondrial ridges disappeared entirely and the mitochondrial membrane was incomplete in the heart of D5w mice following IR (Fig. 6). All these phenomena pointed out that the D5w diabetic hearts underwent severe ferroptosis when facing IRI.

### The Effects of NAC on Post-ischemic Myocardial Ferroptosis, Infarct Size, and Cardiac Ultrastructure in Diabetic Mice

We have previously shown that NAC confers cardio protection against diabetic MIRI primarily through inhibiting excessive autophagy [33]. In the meantime, ferroptosis is a type of autophagy-dependent cell death [42]. Thus, we determined whether NAC could also protect diabetic hearts against IRI through inhibiting abnormal ferroptosis. After NAC treatment, food consumption and water intake were significantly reduced compared to the diabetic group (all *p* < 0.05, Table 1). Body weight in diabetic mice was significantly reduced, and NAC had no significant impact on that. As expected, the degree of myocardial protection begins after 1 week of NAC treatment and becomes more pronounced after 4 week’ treatment. As shown in Figs. 3, 4, and 5, NAC treatment had a certain inhibitory effect on ferroptosis under diabetes mellitus at baseline conditions. CK-MB is considered a diagnostic marker for evaluating post-ischemic myocardial cellular injury to predict infarct size. In a mouse model, we found that CK-MB significantly increased after IR in different groups, which was associated with various degrees of post-ischemic myocardial infarction. As shown in this study, when the mice were subjected to IRI, NAC treatment for 1 week slightly but significantly reduced the post-ischemic myocardial infarct size (*p* < 0.05). However, NAC treatment for 4 week conferred the most profound protection against IRI, evidenced as significant smaller post-ischemic infarct size compared to either D5w + IR or even D2w + IR + NAC (all *p* < 0.05) (Fig. 5A). Meanwhile, post-ischemic plasma CK-MB level, 15-F2t-IsoP level, MDA level, and labile iron level were also significantly lower compared to D5w untreated group (*p* < 0.05). The western blotting and IHC results showed that the levels of Gpx4, Slc7a11, and ferritin had been upregulated by NAC as well (*p* < 0.05), and the effects of 4 week NAC treatments were better than that of only 1 week NAC treatment. Post-ischemic myocardial iron infiltration was also significantly ameliorated by NAC as reflected by Prussian blue staining (Fig. 3G, 4G). As for cardiac ultrastructure, NAC treatment did not completely prevent the destruction of mitochondria, but it did restore some mitochondrial cristae and membrane structure, and the number of lipid droplets and the degree of oxidation were reduced (Fig. 6; Table 2). By prior application of Erastin, we further confirmed that myocardial protection by NAC in diabetic mice is achieved majorly or in part by inhibiting ferroptosis (Fig. 5A–C). Interestingly, we found that the post-ischemic levels of ferroptosis protein, infarct size, CK-MB, cardiac function and morphological microstructure between D2w mice and non-diabetic mice did not significantly differ after 1 week NAC treatment (Fig. 5; Supplementary Fig. 4). These results are supportive of our hypothesis that ferroptosis is just initiated in D2w when the cardiac injury is not that severe, and, as such NAC protected the heart primarily through its antioxidant capacity rather than or to a much less degree through anti-ferroptosis. When time comes to the week 5 diabetes, the protective effect of NAC is attributable to the inhibition of ferroptosis pathway.

**Table 2:** Morphological changes of myocardial ultrastructure

Group/characteristic alterations	Vacuolization of mitochondria	Rupture of mitochondria	Lipid droplets	Total injury score
Normal control	1	1	5	7 ± 2
NC + IR	12	17	11	40 ± 3*
D1w + IR	10	14	15	39 ± 2*
D2w + IR	15	18	17	50 ± 3*#
D5w + IR	28	32	28	88 ± 6*#
D2w + NAC + IR	14	17	16	47 ± 3*#
D5w + NAC + IR	20	23	19	62 ± 2*#^
D5w + NAC + IR + Erastin	26	29	24	79 ± 4*#$

Statistics of characteristic alterations of ferroptosis under ultrastructure of myocardium. The data in the Characteristic alterations were the quantities in the schematic figure, while those in the Total are the results of counting 5 different sets of views and expressed as mean ± SD

*D1w* Diabetes for a week, *D2w* Diabetes for 2 week, *D5w* Diabetes for 5 week, *D2w* + *NAC* Diabetes for 2 week and NAC treatment for 1 week, *D5w* + *NAC* Diabetes for 5 week and NAC treatment for 4 week

*p < 0.05 versus control, ^#^*p* < 0.05 versus NC + IR, ^^^*p* < 0.05 versus D5w + IR, ^$^*p* < 0.05 versus D5w + NAC + IR

## Discussion

Diabetes is a metabolic disease characterized by the body's inability to maintain normal glucose homeostasis. It represents a significant threat to human health and is one of the leading causes of increased morbidity and mortality worldwide. According to the International Diabetes Federation, there are 415 million adults living with diabetes, with the number expected to rise to over 642 million by 2040 [43]. This indicates that diabetes and its complications are among the most severe health challenges faced globally. Diabetes exists in several forms, with Type 1 diabetes, Type 2 diabetes, and gestational diabetes being the most common [44]. Epidemiological studies of diabetes have shown some gender differences, with a slight male predominance reported in Type 1 diabetes [45]. Diabetics face an increased risk of developing a variety of acute or chronic complications, including diabetic nephropathy, retinopathy, and neuropathy, as well as cardiovascular complications such as coronary artery disease, cardiomyopathy, stroke, and peripheral arterial disease [46]. Cardiovascular complications are among the leading causes of death in diabetic patients [47].

Hearts from subjects with diabetes are vulnerable to MIRI, and recent experimental studies showed that ferroptosis, a type of nonapoptotic, iron-dependent form of cell death, is attributable to myocardial IRI in diabetes [25, 27]. However, studies also showed that hearts from diabetic rodents at the very early stage of diabetes can be more resistant to ischemic insult [48–51], while whether or not the extent of ferroptosis plays a role in determining the myocardial vulnerability to ischemic insult has yet to be explored. In the current study, we examined the myocardial vulnerability to IRI at different stages of diabetes and explored the potential relevance or association of myocardial vulnerability with the extent of ferroptosis in the myocardium. Our current findings show that under natural conditions of diabetes, especially before D5w, D1w as well as D2w exhibited no or little change in ferroptosis-related proteins such as Gpx4 and Slc7a11, while at D5w this change became pronounced. In D1w, there exists enhanced resistance to myocardial IRI, while D5w aggravated myocardial injury. In addition, we applied NAC for short-term as well as long-term treatment, and showed that long-term treatment reversed myocardial IRI after D5w, and its protective effect could be reversed by Erastin.

Oxidative stress is an important mechanism in MIRI [10, 52]. When the myocardium is hit by IR, the high production of ROS during the ischemic phase as well as during the reperfusion phase in the absence of sufficient endogenous antioxidant capacity of the myocardium lead to myocardial tissue damage. Massive ROS production leads to cellular oxidative damage accompanied by a decrease in glutathione peroxidase [53]. Diabetes further increases harmful ROS and inhibits glutathione peroxidase, greatly exacerbating myocardial tissue damage [54]. At 1 week of diabetes induction, we found that GSH levels were elevated in diabetic mice. Moreover, we found that free iron was reduced in the infarct region after IRI in early diabetes compared to controls. And this effect was correlated with ferritin. This suggests that there is indeed an enhanced antioxidant capacity in D1w to resist IRI. However, this compensatory protective effect became weakened or lost over time. At 2 week of diabetes, the myocardium of diabetic mice started to show damage and a large amount of iron deposition was found in the infarcted region. The presence of large amounts of iron deposition in the myocardial ischemic zone in the presence of increased intracellular ROS level, lead to exacerbated myocardial injury. At 5 week of diabetes, the diabetic mice hearts also demonstrated decreased GSH and ferritin, and these factors promoted lipid peroxidation formation manifested as increased MDA and 15-F2t-IsoP. This in turn induced the development of ferroptosis and increased infarct size as well as CK-MB. Short-term NAC treatment initiated at the early phase of diabetes could manifest its myocardial protective effects primarily through its antioxidant properties since ferroptosis is just initiated in D2w when the cardiac injury is not that severe. 5 week later, 15-F2t-IsoP and MDA levels were significantly increased in diabetic mice, and myocardial damage became severe, especially after IR that was accompanied with significant increase in ferroptosis. At this point, NAC conferred its cardioprotective effects against IRI in diabetic mice primarily though attenuating ferroptosis given that the application of Erastin reverted its protective effects. Of note, there was no significant change in cardiac fibrosis after IR, regardless of whether diabetes was present or not, or whether it was treated with NAC (Supplementary Figs. 1, 2), the reason could be that transient ischemia–reperfusion injury is not sufficient to affect myocardial fibrosis, even in diabetes. The novel finding of the current study is that increased ferroptosis in the myocardium of D5w mice may be the main mechanism that makes the diabetic heart more susceptible to ischemic injury than the hearts of non-diabetic subjects, and that NAC treatment-mediated attenuation of diabetic MIRI is attributable to the reduction of ferroptosis.

Ferroptosis is an iron-dependent, novel form of programmed cell death that is distinct from apoptosis, cell necrosis, and cell autophagy [21]. The main mechanism of ferroptosis is the formation of lipid peroxides, the accumulation of which in the cell membrane eventually disrupts the membrane integrity and thus induces cell death. Ferroptosis also is manifested by a decrease in the regulatory core enzyme Gpx4 of the antioxidant system (glutathione system). Free Fe2 + after MIRI catalyzes the generation of -OH from H2O2. The presence of -OH radicals, which eventually participate in the Fenton reaction, can promote the production of ROS. This triggers oxidative damage to nearby biomolecules (e.g., proteins, DNA, and lipids). Our experimental results show that at D5w, lipid peroxidation is significantly increased in myocardial tissue after IR induction. In addition, the expression of significantly increased free iron was increased in myocardial tissues after IR induction. The expression of proteins related to the inhibition of tissue ferroptosis was also significantly decreased. The results indicated that myocardial tissue ferroptosis was increased in mice after IR induction.

NAC has free radical scavenging properties and can act as a broadly active antioxidant to inhibit cell death by blocking ROS and the subsequent lipid peroxidation [31–33]. It has been shown that NAC further attenuates mitochondrial oxidative damage and ferroptosis by enhancing mitochondrial GSH activity and maintaining mitochondrial redox homeostasis [55]. The role of NAC in the mechanism of ferroptosis during MIRI injury remains largely unclear. In our experiments, we found that NAC applied to diabetic mice significantly reduced myocardial histopathological damage, decreased free iron in myocardial tissue, significantly reduced ferroptosis through enhancing related ferroptosis inhibition proteins, and decreased MDA, 15-F2t-IsoP, and lipid peroxidation levels after IR. Thus, inhibition of ferroptosis may represent a major mechanism by which NAC attenuate myocardial IRI in diabetic mice.

Gpx4, one of the glutathione peroxidases, is an important antioxidant enzyme in vivo [56]. Gpx4 is involved in the reduction reaction of intracellular lipid peroxidation, reducing cytotoxic lipid peroxides to the corresponding alcohols. Gpx4 is the most important intracellular anti-lipid peroxidase and an important regulator of ferroptosis [56, 57]. It has been demonstrated that Gpx4 and its downstream target genes are downregulated in cardiomyocytes of diabetic rats, which exacerbates diabetic myocardial dysfunction, hypertrophy, and inflammatory responses, and exacerbates MIRI in diabetic rats [25, 58]. In addition, ferritin is a key protein for maintaining iron homeostasis. As a key molecule that promotes ferritin phagocytosis, ferritin plays a negative regulatory role in iron phagocytosis in cardiomyocytes [26]. It was shown that iron supplementation in ferritin-deficient mice caused myocardial ferroptosis by inhibiting Slc7a11-mediated glutathione production [59]. In our experiments, we found increased protein expression of Slc7a11, Gpx4, and ferritin after NAC application to diabetic mice, indicating the activation of the System xc-/Gpx4 axis. Erastin is a small molecule that induces ferroptosis through the system xc-/Gpx4 mechanism. Thus, by injecting Erastin into diabetic mice, we found that Erastin reversed the protective effect of NAC against myocardial IRI in diabetic mice.

In summary, our study demonstrates that during the development of early diabetes in mice, susceptibility to cardiac IRI emerges at approximately 5 week, that the mechanism may be related to the activation of ferroptosis. Treatment with the antioxidant NAC could partially and significantly restore the impairment in cardiac function in diabetes by inhibiting abnormal cardiac ferroptosis and prolonged NAC treatment may confer better cardioprotective effects. Moreover, further work is needed to fully understand the mechanisms governing NAC mediated inhibition of myocardial ferroptosis in diabetes and to explore its potential clinical implications. This includes investigating myocardial ferroptosis and assessing NAC treatment in animals with longer durations of diabetes, and mining the factors contributing to the reduction of GPX4 or Slc7a11 in the early stages of diabetes.

### Supplementary Information

Below is the link to the electronic supplementary material.

Supplementary file1 (PDF 1314 kb)

Supplementary file2 (PDF 1450 kb)

## Data Availability

The data used to support the findings of this study are available from the corresponding author upon request.

## Abbreviations

IRI: Ischemia–reperfusion injury
Gpx4: Glutathione peroxidase 4
NAC: N-acetylcysteine
DM: Diabetes mellitus
MIRI: Myocardial ischemia–reperfusion injury
AMI: Acute myocardial infarction
ROS: Reactive oxygen species
STZ: Streptozotocin
GSH: Glutathione
LAD: Left anterior descending coronary artery
TTC: 2,3,5-Triphenyltetrazolium chloride
AAR: Area at risk
IS: Infarct size
CK-MB: Creatine kinase MB
MDA: Malondialdehyde
15-F2t-IsoP: 15-F2t-Isoprostane
HE: Hematoxylin and eosin
ABC: Avidin–biotin peroxidase complex
IHC: Immunohistochemistry
ACSL4: Acyl-CoA synthetase long-chain family member 4
