# Supplementary material for: N-acetylcysteine Protects Against Myocardial Ischemia–Reperfusion Injury Through Anti-ferroptosis in Type 1 Diabetic Mice
Source: Cardiovasc Toxicol. 2024 Apr 22;24(5):481–98. doi: 10.1007/s12012-024-09852-7 (PMC11076402; doi:10.1007/s12012-024-09852-7)
Supplement: Supplementary file 1 — Supplementary file1 (PDF 1314 kb) [file 12012_2024_9852_MOESM1_ESM.pdf]

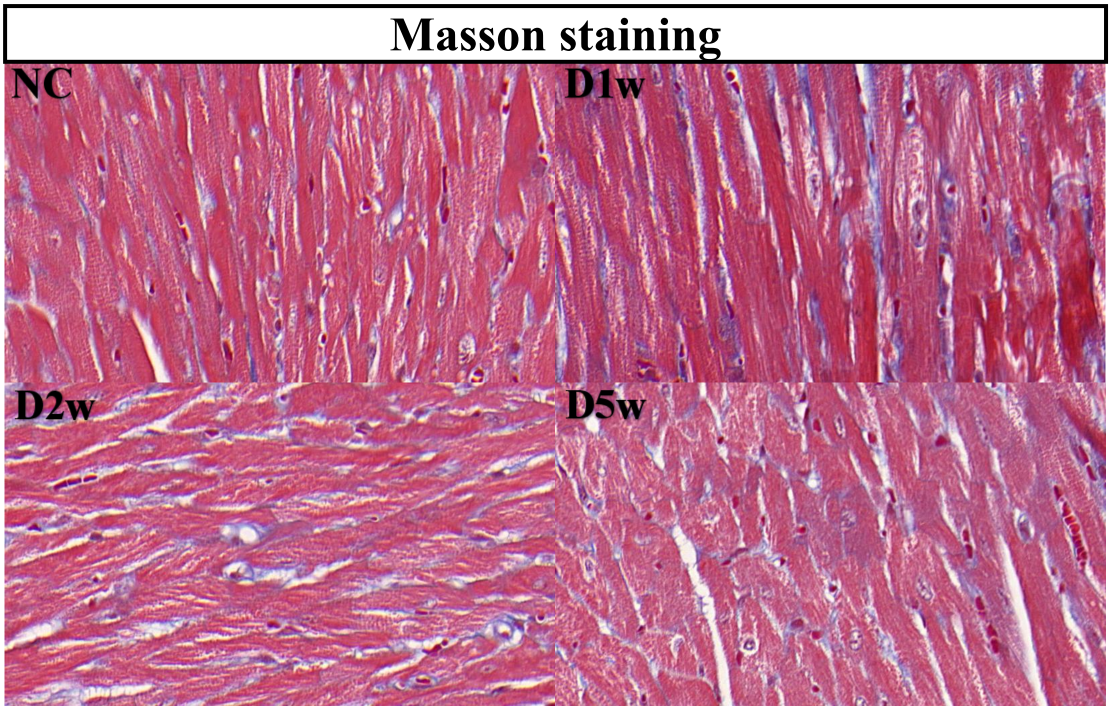

Supplemental Figure 1. Masson staining of myocardium in diabetic mice without IR. Masson's staining was performed and the myocardial collagen fibers were stained in blue, while the myocardial cells were stained in red. There were no effect on myocardial fibrosis in the baseline of diabetes mellitus, even in D5w.

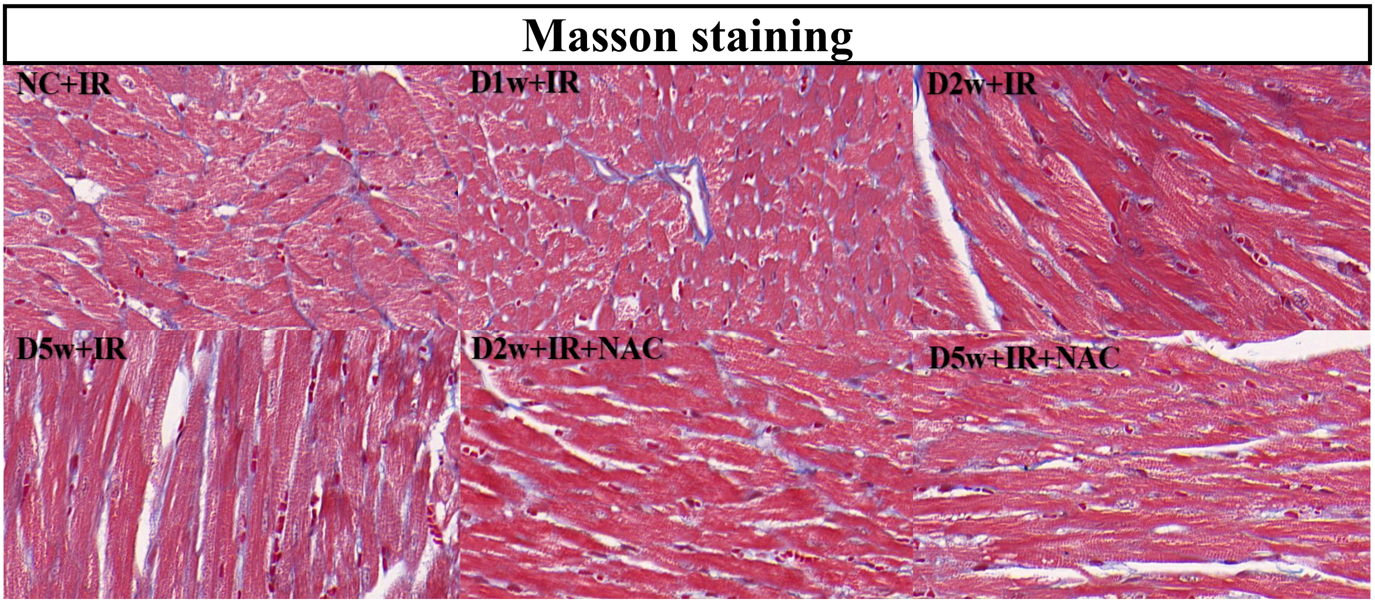

Supplemental Figure 2. Masson staining of myocardium in diabetic mice with IR. After IR, the positive sign of collagen fibers were increased but not significant and presented Masson-stained myocardial section was similar among groups, which could be explained by the reason that transient ischemia-reperfusion injury is not sufficient to affect myocardial fibrosis, even in diabetes.

A

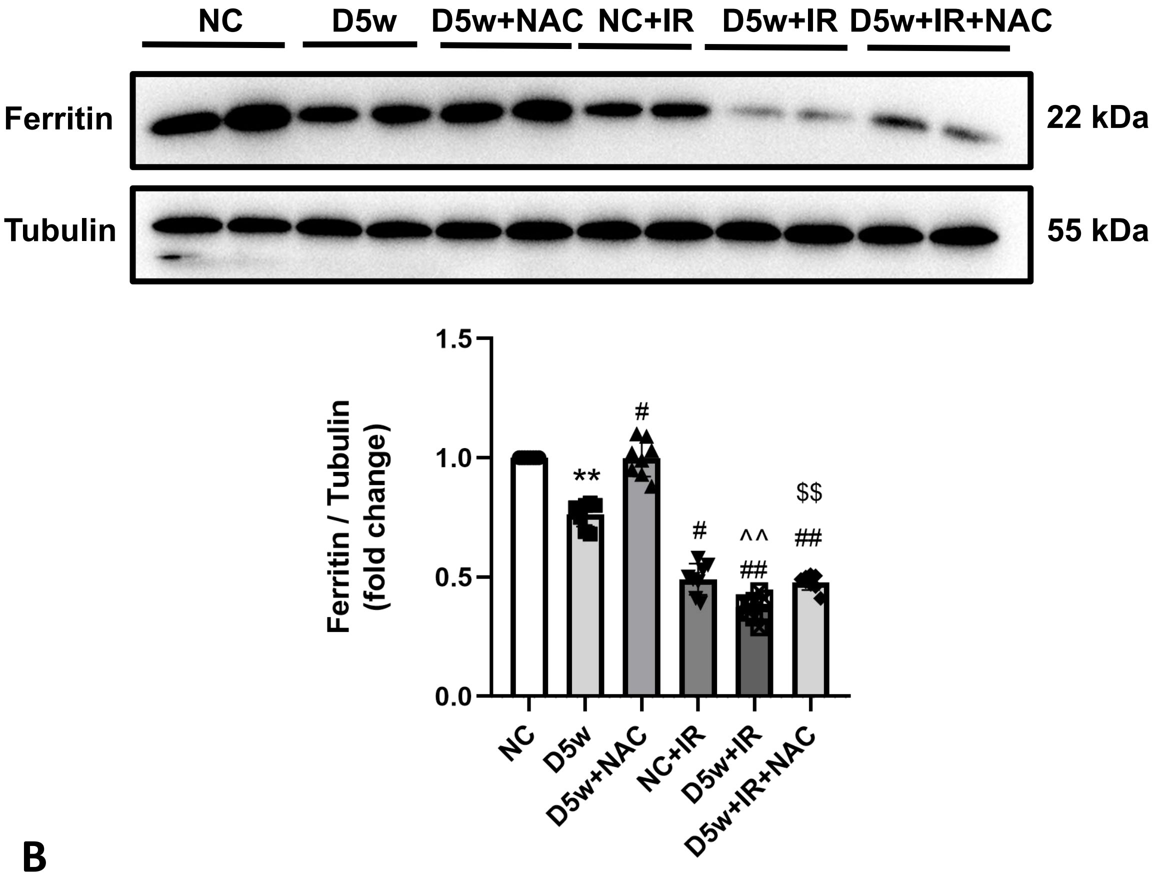

B

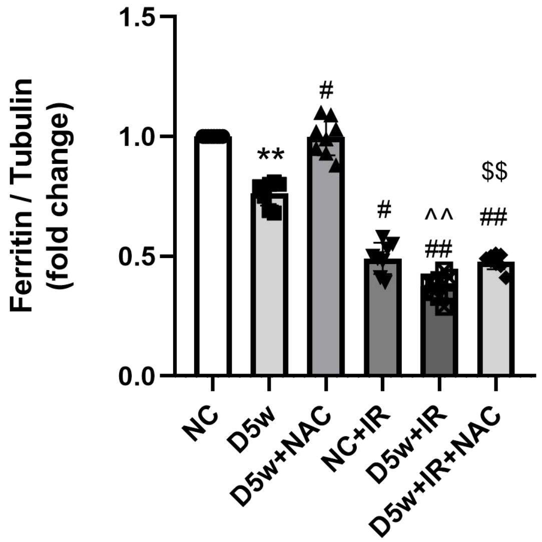

Supplemental Figure 3. Expression of Ferritin in D5w mice.

A,B. Due to the high background of IHC of Ferritin, we additionaly performed Western blotting **(A)** and qPCR**(B)** to detect the expression of Ferritin after IR and NAC treament in D5w mice. The results were in accordance with the IHC result and showed that Ferritin was down regulated in D5w and further decreased after IR. NAC treament for 4weeks reversed this abnormal downregulation of ferritin. **p*<0.05, ***p*<0.01, versus NC group; $^{\#}p<0.05$, $^{\#\#}p<0.01$, versus D5w group; ^*p*<0.05, ^^*p*<0.01, versus NC+IR group; $^{\$}p<0.05$, $^{\$\$}p<0.01$, versus D5w+IR.

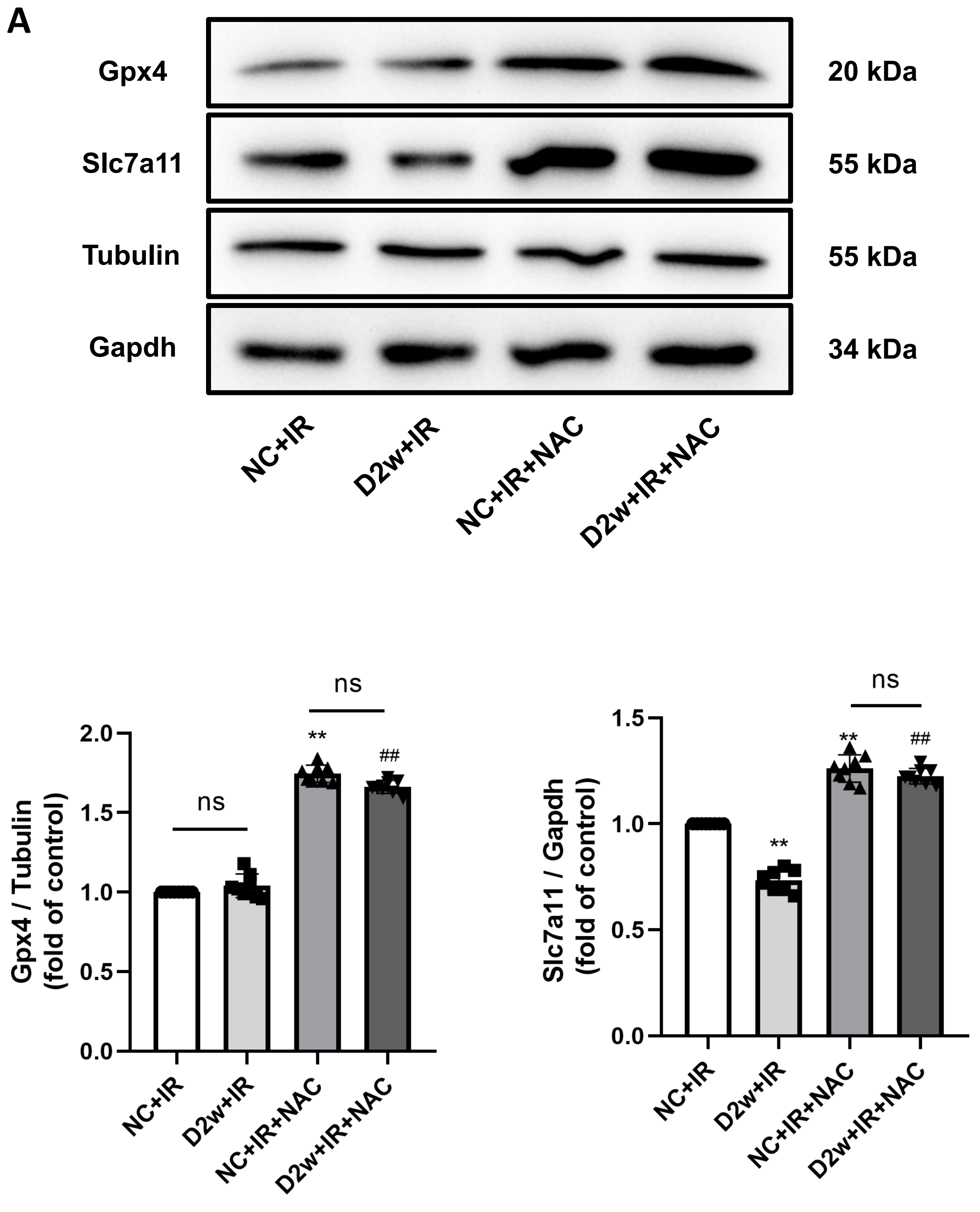

Supplemental Figure 4. Nac treament for 1week has no effect to non-diabetic mice.
A. Expression of Gpx4 and Slc7a11 in DM mice was assessed using western blotting. The results showed that there was no significant difference between the NC+IR+NAC group and the D2w+NAC+IR group. *p<0.05 **p<0.01, versus NC+IR group; #p<0.05 ##p<0.01, versus D2w+IR group.

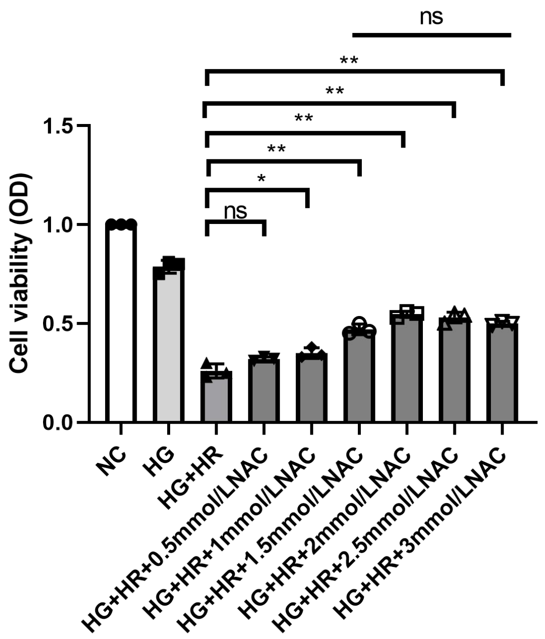

Supplemental Figure 5. Cell viability of different NAC treament in H9C2.

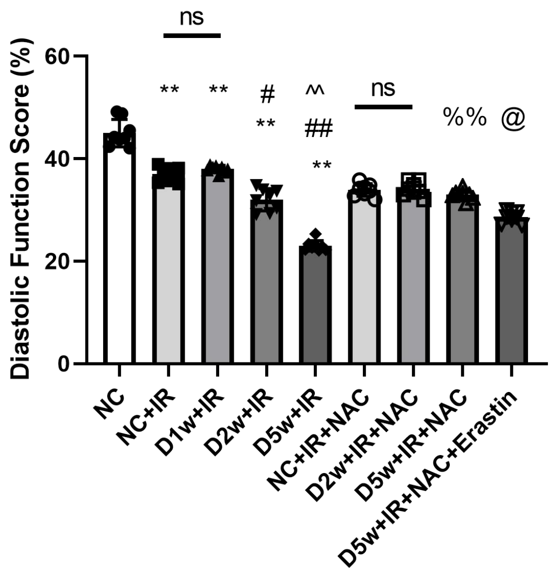

Supplemental Figure 6 . Diastolic funtion of diabetic mice upon IR with or without NAC treatment.
