# Supplementary material for: N-acetylcysteine Protects Against Myocardial Ischemia–Reperfusion Injury Through Anti-ferroptosis in Type 1 Diabetic Mice
Source: Cardiovasc Toxicol. 2024 Apr 22;24(5):481–98. doi: 10.1007/s12012-024-09852-7 (PMC11076402; doi:10.1007/s12012-024-09852-7)

Whole membrane WB image for Gpx4 and Slc7a11 in Figure 1

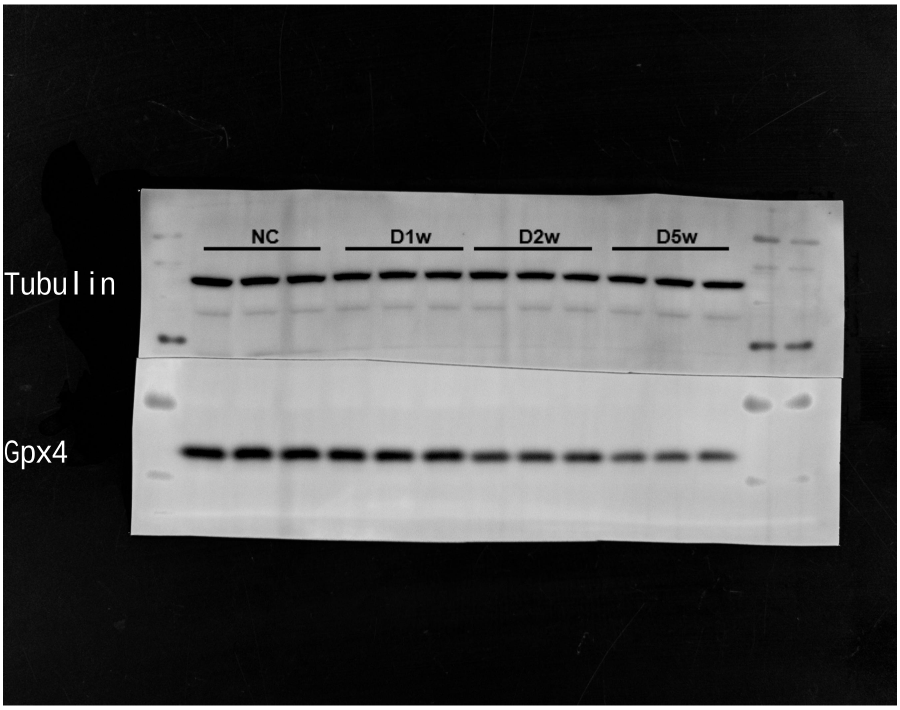

75 kDa

25 kDa

Slc7a11

Gapdh

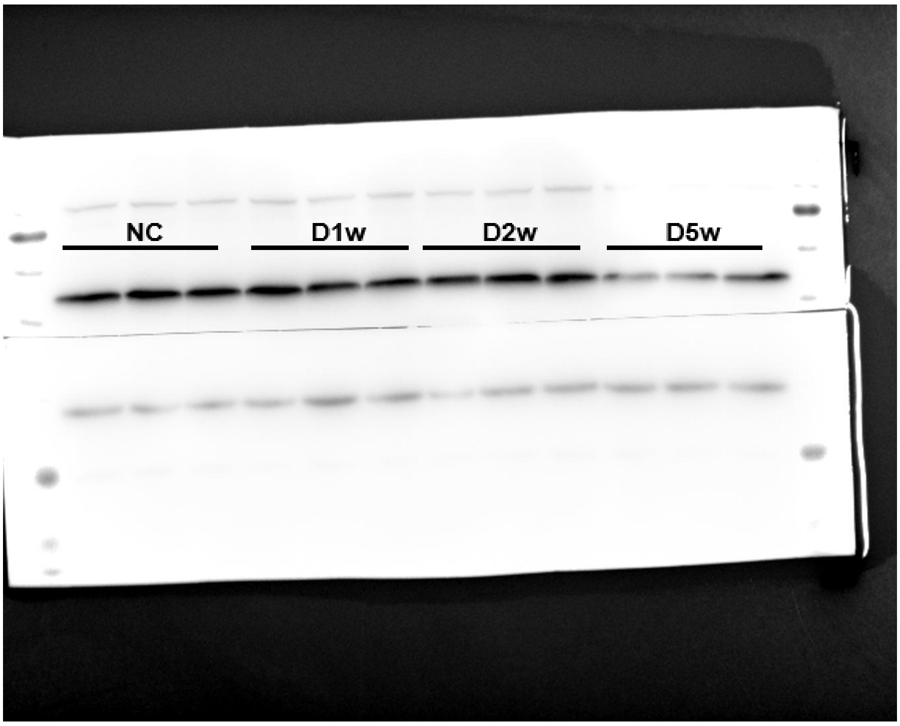

75 kDa

shorter time of exposure

25 kDa

Slc7a11

Gapdh

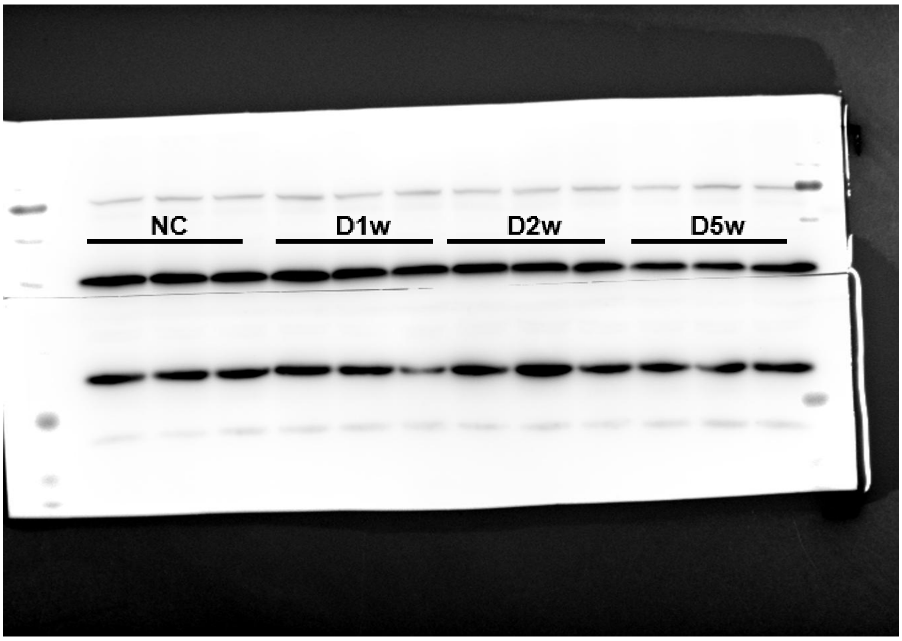

75 kDa

longer time of exposure

25 kDa

Whole membrane WB image for
Gpx4 and Slc7a11 in Figure 2

Tubulin

Gpx4

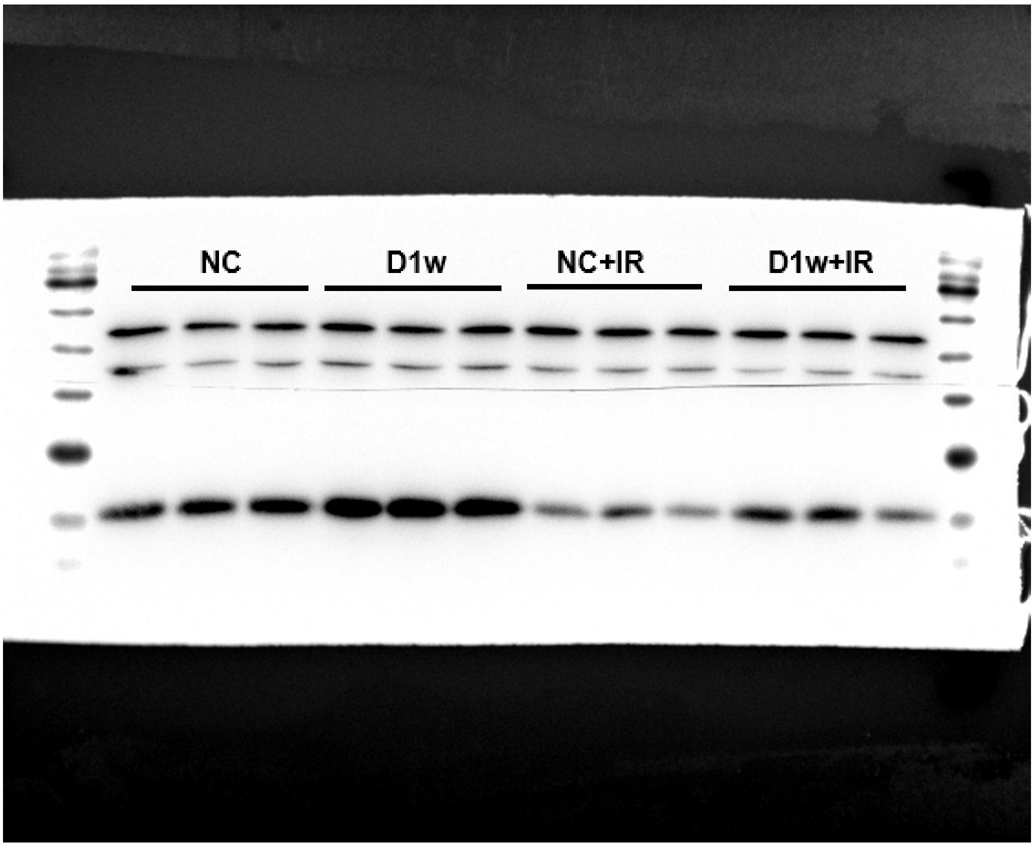

75 kDa

25 kDa

Slc7a11

Gapdh

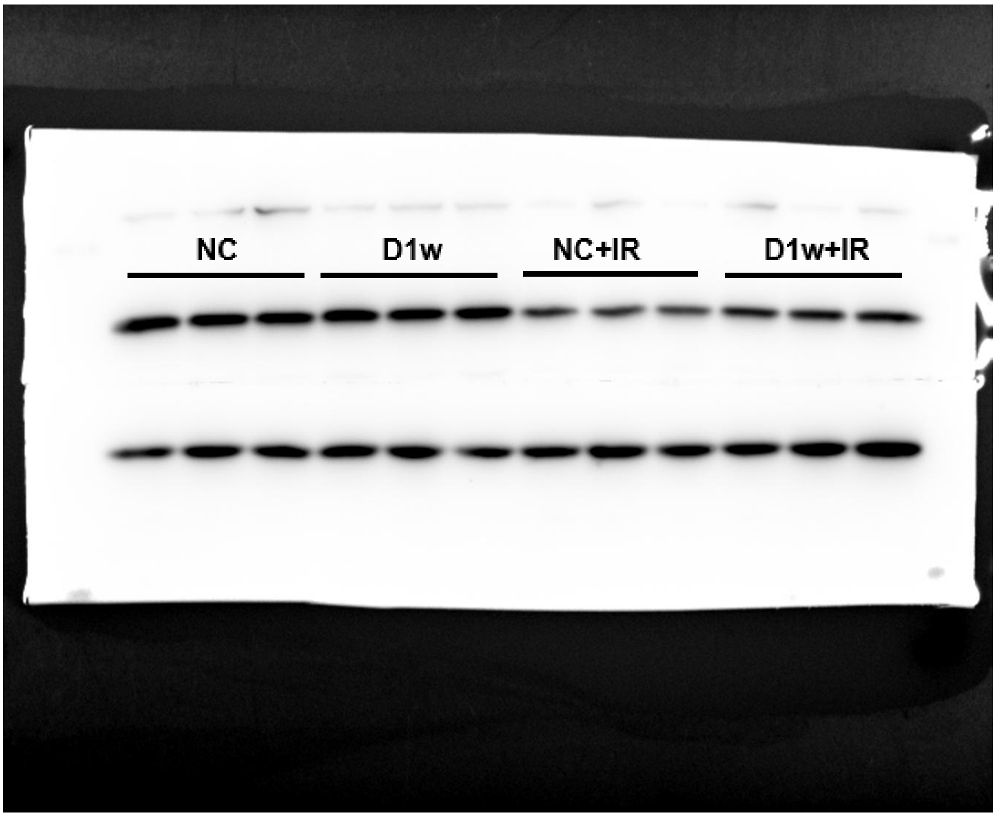

75 kDa

25 kDa

Whole membrane WB image for
Gpx4 and Slc7a11 in Figure 3

Tubulin

Gpx4

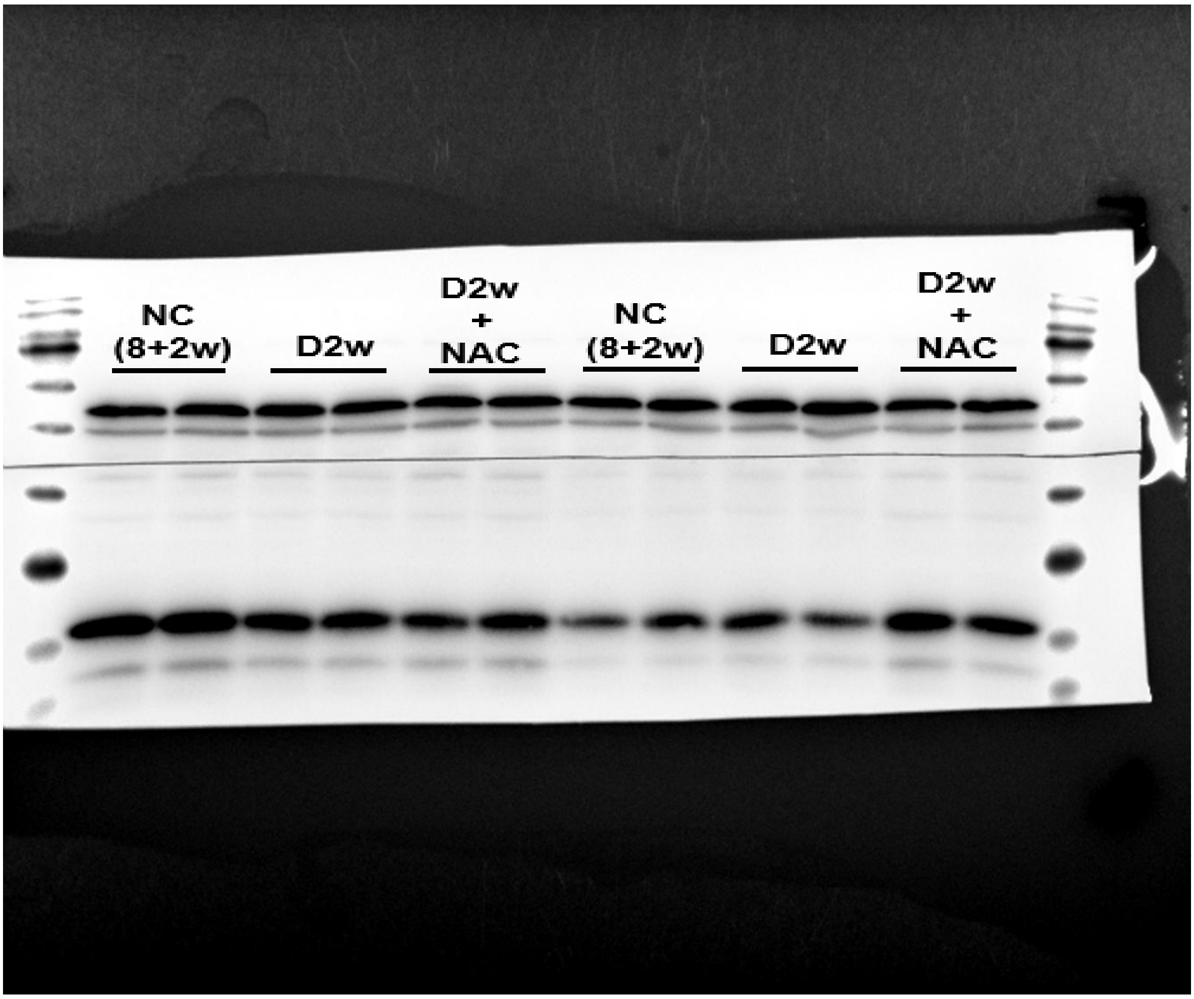

75 kDa

25 kDa

Slc7a11

Gapdh

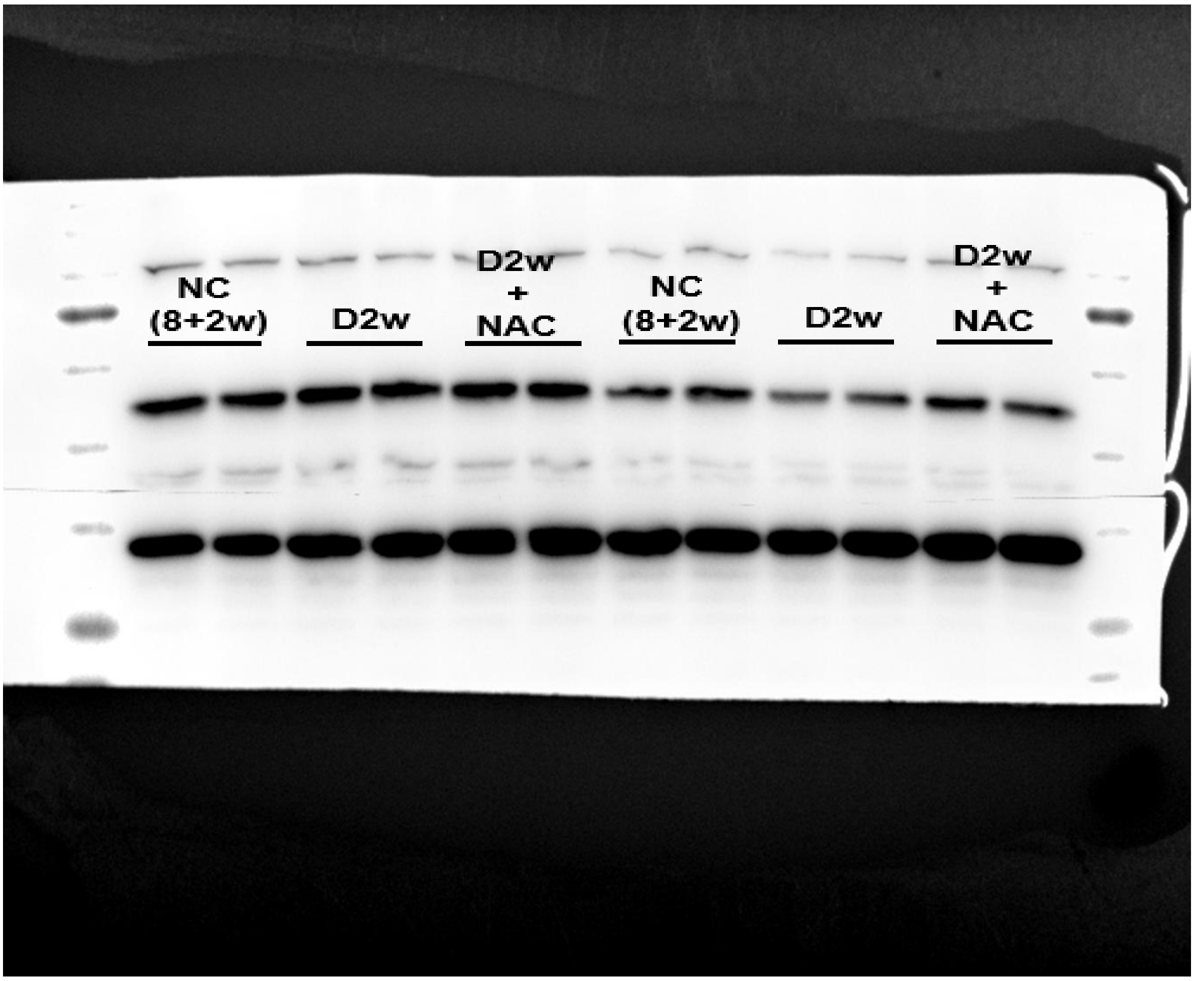

75 kDa

25 kDa

Whole membrane WB image for Gpx4 and Slc7a11 in Figure 4

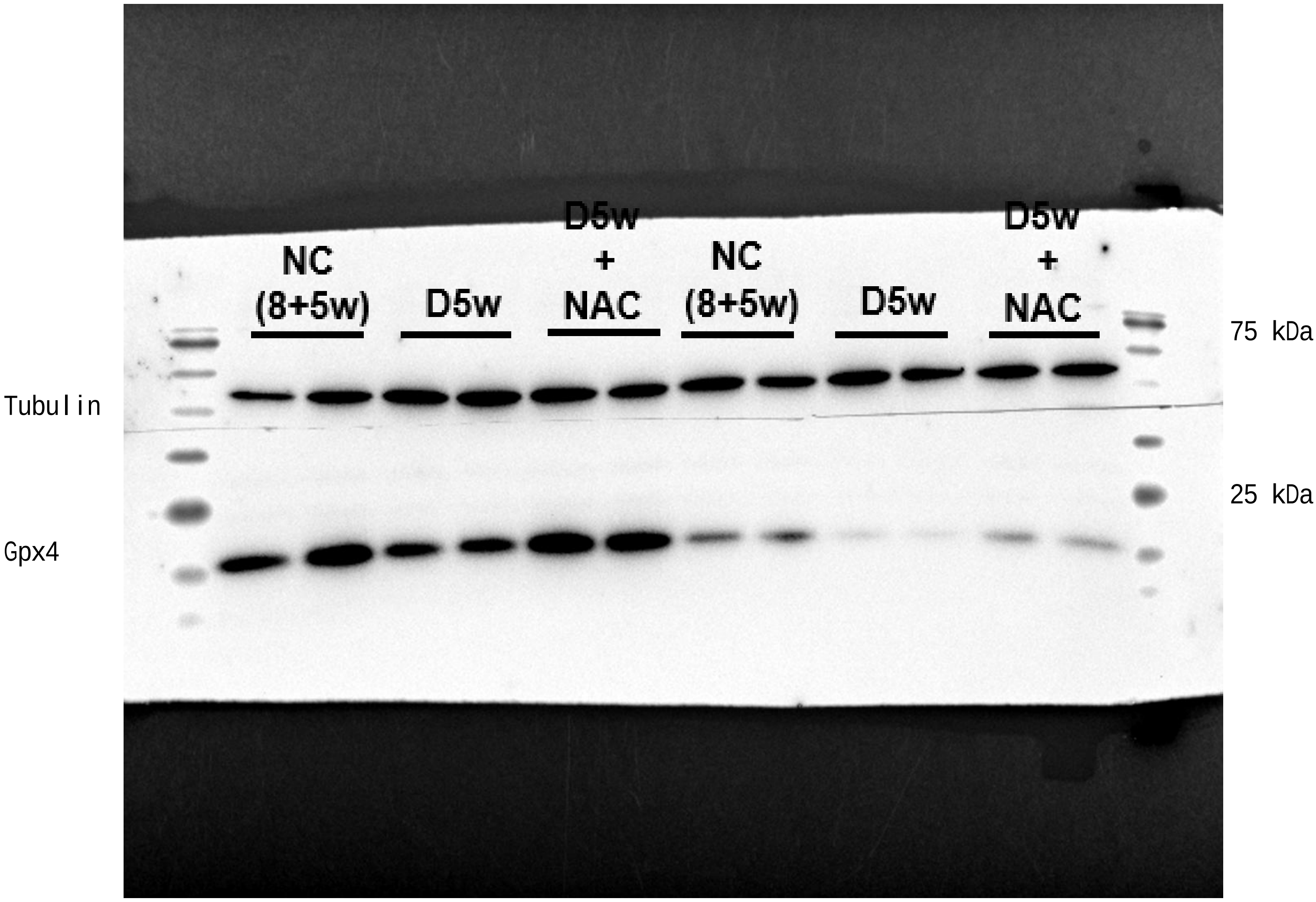

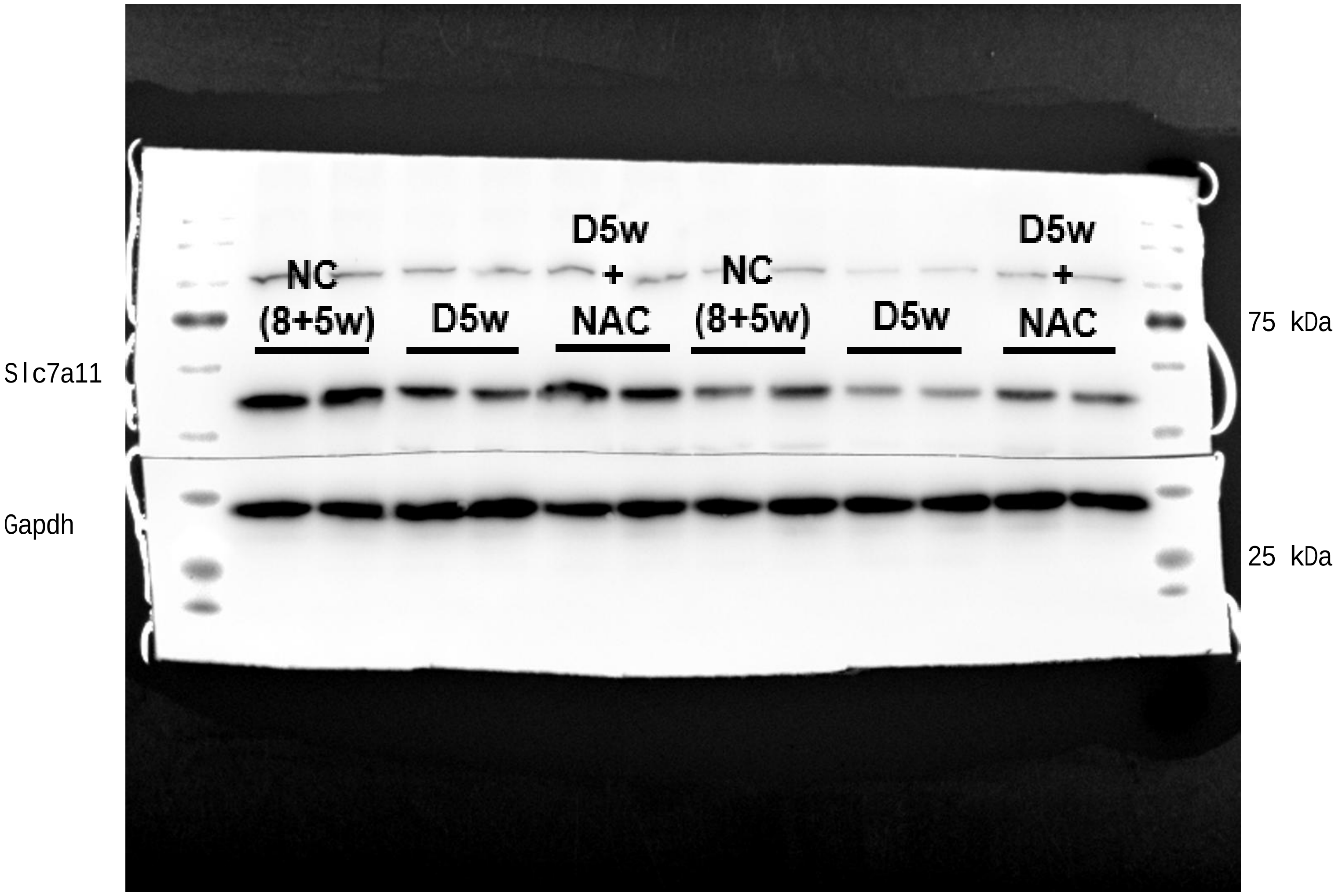

Whole membrane WB image for Ferritin in Supplementary Figure 3

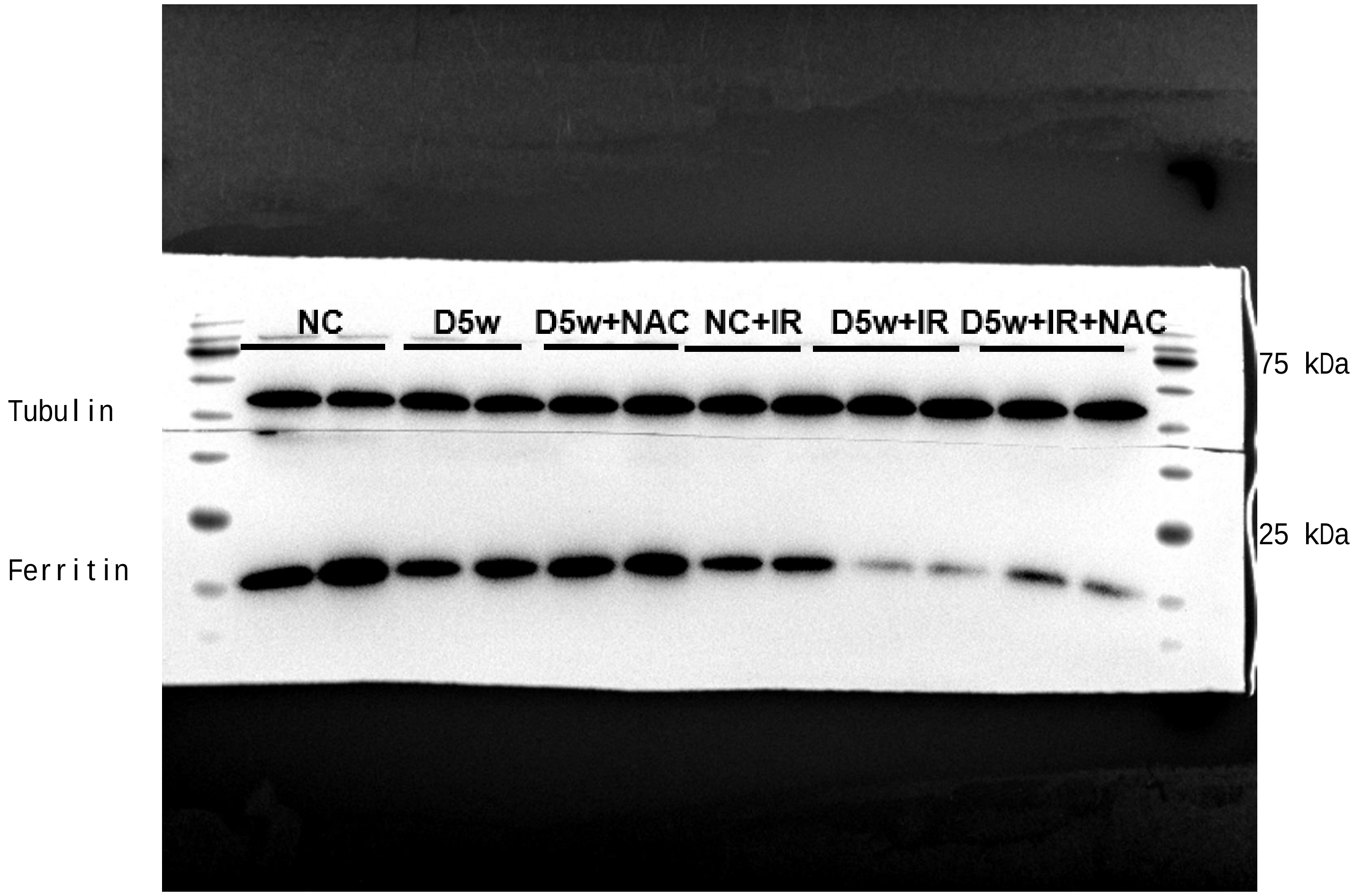

Whole membrane WB image for Gpx4 and Slc7a11 in Supplementary Figure 4

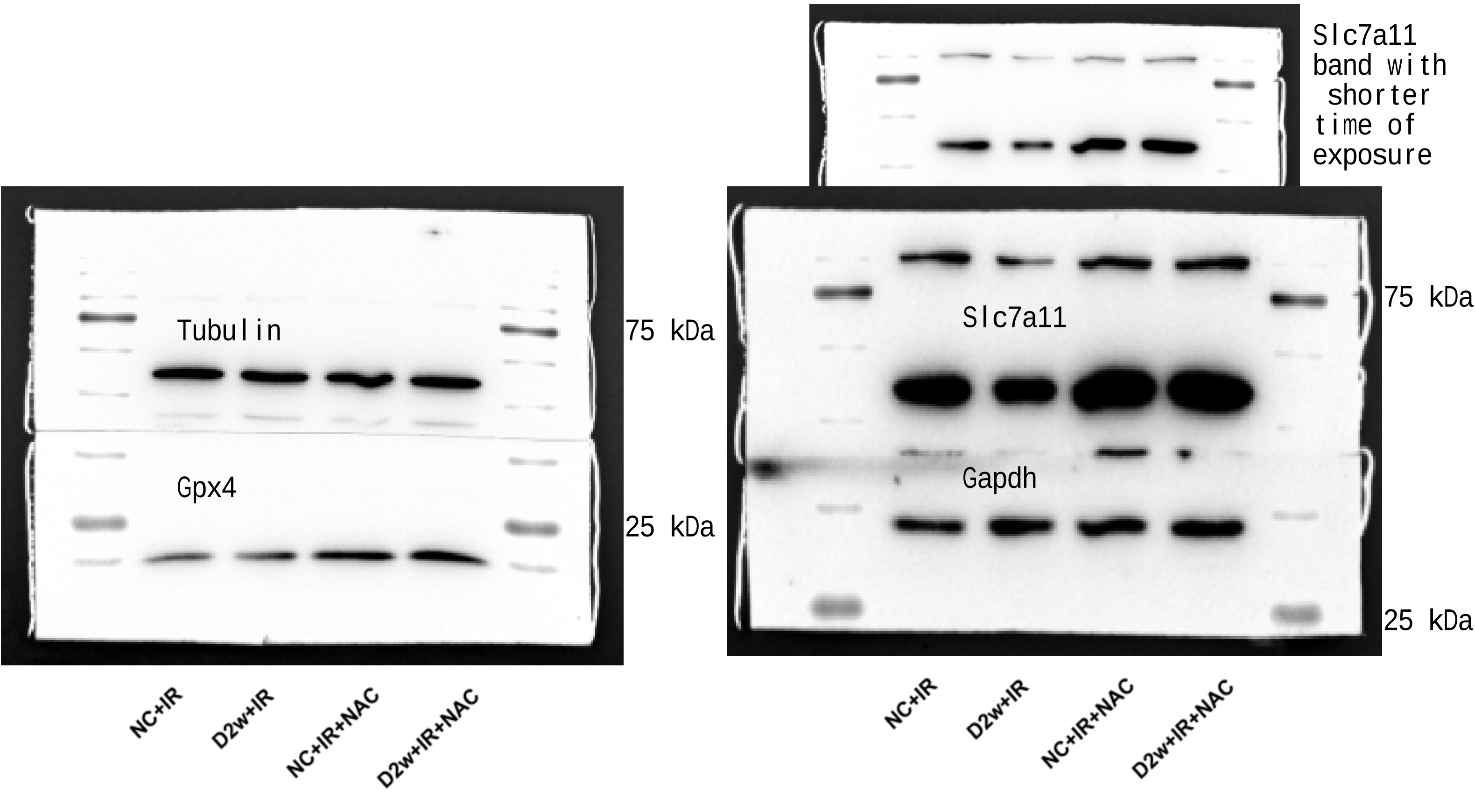

Supplement: Supplementary file 2 — Supplementary file2 (PDF 1450 kb) [file 12012_2024_9852_MOESM2_ESM.pdf]
